# Material and Carbon Footprints of Machinery Capital

**DOI:** 10.1021/acs.est.3c06180

**Published:** 2023-11-22

**Authors:** Meng Jiang, Ranran Wang, Richard Wood, Kajwan Rasul, Bing Zhu, Edgar Hertwich

**Affiliations:** † Department of Energy and Process Engineering, Norwegian University of Science and Technology, Trondheim 7491, Norway; ‡ Institute of Environmental Sciences (CML), Leiden University, Einsteinweg 2, 2333 CC Leiden, The Netherlands; § Department of Chemical Engineering, Tsinghua University, Beijing 100084, China

**Keywords:** machines, industrial equipment, gross fixed capital formation, investment, material stock, capital stock, mass flow analysis, multiregional input–output model, circular economy, socioeconomic metabolism, material footprint

## Abstract

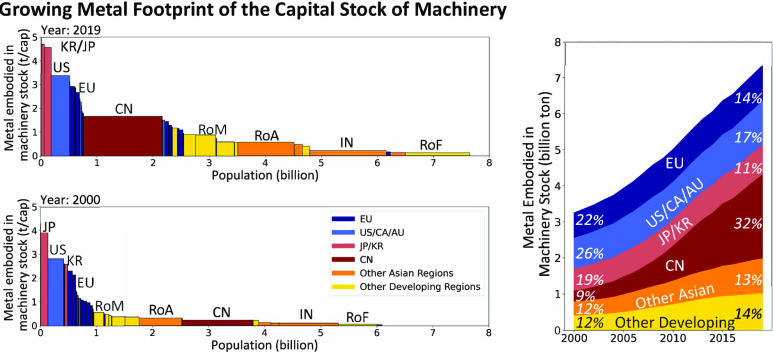

Machinery and equipment, integral as technology-specific capital goods, play a dual role in climate change: it acts as both a mitigator and an exacerbator due to its carbon-intensive life cycle. Despite their importance, current climate mitigation analyses often overlook these items, leaving a gap in comprehensive analyses of their material stock and environmental impacts. To address this, our research integrates input–output analysis (IOA) with dynamic material flow analysis (d-MFA) to assess the carbon and material footprints of machinery. It finds that in 2019, machinery production required 30% of global metal production and 8% of global carbon emissions. Between 2000 and 2019, the metal footprint of the stock of machinery grew twice as fast as the economy. To illustrate the global implications and scale, we spotlight key countries. China’s rise in machinery material stock is noteworthy, surpassing the United States in 2008 in total amount and achieving half of the US per capita level by 2019. Our study also contrasts economic depreciation—a value-centric metric—with the tangible lifespan of machinery, revealing how much the physical size of the capital stock exceeds its book values. As physical machinery stocks saturate, new machinery can increasingly be built from metals recycled from retired machinery.

## Abstract 2

Industrialized countries use 2−4.5 tons of metals per capita to build their stock of machinery and equipment, while many developing countries dispose only of a tenth of that amount.

## Introduction

Our industrial society is built upon the foundation of machinery capital, encompassing the vast array of tools essential for industries—including industrial robots, logistics equipment, agricultural machinery, and electricity grids.^1^ These tools efficiently produce goods and provide services. From daily essentials such as commercial washing machines to advanced technological devices such as graphics processors, machinery has woven itself into the fabric of both industrial processes and daily life. As we witness a trend toward automation and robotics, even service industries are seeing an uptick in machinery use. Further, the shift toward a sustainable energy system and circular economy accentuates the role of machinery like wind turbines, solar panels, batteries, and waste-sorting robotics.^2^ Given machinery’s ubiquitous presence, understanding its broader impacts is crucial.

Machinery and equipment fall under the important category of “manufactured capital” accumulating in the industrial system.^3^ However, there is a growing concern about their stock scales and environmental impacts. They not only act as critical infrastructure for climate mitigation technologies and service provision^3−8^ but also possess a carbon-intensive life cycle during their construction and operation phases.^9−11^ Notably, while extensive research focuses on economy-wide capital or particular assets like buildings^12−15^ and vehicles,^13,15−17^ machinery and equipment often remain overlooked (except for some pieces of electricity infrastructure^18,19^). This is despite the fact that they constituted the second-largest stock of metal in the economy next to buildings and infrastructure.^9,20^ For perspective, the production of machinery accounted for 8% of global greenhouse gas emissions in 2015^21^—outpacing the emissions of aviation and ocean freight combined. This significant oversight highlights a critical knowledge gap.

While various material- or climate-related models exist, they often do not adequately represent machinery and equipment. Dynamic material flow analysis (d-MFA) models,^22^ for instance, tend to emphasize economy-wide material stocks^9,23^ overlooking machinery and equipment. Environmentally Extended Input–Output Analysis (EEIOA) links material production and consumption. Still, recent IO-based analyses on capital stocks have not been tailored to machinery specifically.^3,4,6,10,24,25^

Many studies incorporated (endogenized) capital as a production input within the Input–Output Analysis (IOA) framework.^4,24−26^ This approach is retrospective and considers the environmental impact of capital investments made in the past.^6,27^ However, it is bound by its limitations, as it determines the environmental footprint multiplier based on the year of analysis, not the year the capital was created.^4^ While introducing a dynamic IOA model might refine this approach,^28^ it would add complexity. Alternatively, some research has opted for the dynamic flow concept, allocating the future environmental footprint of Gross Fixed Capital Formation (GFCF) with either capital depreciation^10,29^ or the life span of the physical stock.^3^ However, a noticeable inconsistency arises between these two metrics as assets often outlast their depreciation period. This discrepancy, which could impact scope 3 accounting for businesses, is yet to be adequately addressed in current studies.

Broader economic-energy-environment models such as integrated assessment models (IAM)^30^ for climate mitigation analysis, cover machinery without much detail, if at all.^30,31^ Considering machinery’s pivotal role in industries worldwide, this ambiguity impedes the formulation of accurate environmental mitigation strategies. While specialized engineering-focused studies investigated specific technological assets,^18,19,32^ their scope remains narrow for a comprehensive global analysis of climate change mitigation and resource efficiency measures. Hence, even as manufacturers and users understand the function and specifications of machinery they produce or acquire, the bigger picture of capital stock scale and environmental impacts remains elusive.

Addressing the gap in our understanding of machinery capital and its environmental consequences is vital. This knowledge will aid future research, especially given the central role of machinery in climate change mitigation. Here, we aim to answer the following research questions: How have global and regional trends in machinery stocks evolved in recent decades? What are the environmental impacts of these trends in terms of GHG emissions, materials, and primary metals?

In this study, we leverage the footprints of GHG emissions, materials, and primary metals related to machinery production as proxies to infer global and regional machinery stock trends, particularly in the absence of detailed bottom-up machinery data. We first derived the footprint from annual production processes. This enables us to see the emissions and resource consumption resulting from machinery and equipment production in each region and year. Then, we apply the cohort model to accumulate the footprint of capital formation and retirement each year across time to obtain the stock. Given the predominance of metals in machinery and the consistency of metal processing, the metal footprint serves as a reliable metric for approximating the machinery stock scale. It represents not the actual metal content but the accumulated input of metals in the production of the product (encompassing a global supply chain and cradle-to-gate perspective). Moreover, we underscore the distinctions and implications between capital depreciation and stock life span when allocating environmental footprints.

Central to our study is machinery capital, excluding machinery for household consumption, such as washing machines, as these are accounted for under household expenditures (in economic accounts) and thus fall outside our research scope. Prior work has addressed consumer durables.^33^ Our accompanying footprint results indicate that households and government cause a large share of final demand for “radio, television, and communications equipment and apparatus’ but only very little (mechanical) “machinery and equipment nowhere else classified”; see Figures S1 and S2.

## Methods

In this study, we use a broader definition of the machinery and equipment sector to include a wider range of industrial products. Employing the footprint calculation on the EXIOBASE platform,^34^ we assess environmental flows, covering GHG emissions, material extraction, and primary metal use^35^ from machinery production. These flows feed into stock assessments through dynamic material flow analysis, using survival curve and depreciation methods, respectively, to measure the footprint embodied in machinery stock across regions over time. Capital data sets provide a foundation for understanding machinery’s environmental footprint allocation.

### Calculating Flows

#### Scope of Machinery And Equipment

The machinery and equipment sector supplies equipment essential for mining, manufacturing, energy, and construction, while also producing household appliances.^1^ For our study, we expand the definition^36^ to encompass transportation, furniture, and other goods linked to industrial production. We chose 8 final products from EXIOBASE as the assets related to machinery and equipment, as shown in Table 1.

**Table 1:** Products Related to Machinery and Equipment in EXIOBASE

product no.	product/asset	abbreviation
118	machinery and equipment n.e.c.	GenMach
119	office machinery and computers	IT
120	electrical machinery and apparatus n.e.c.	ElectrMach
121	radio, television, and communication equipment and apparatus	Commn.
122	medical, precision, and optical instruments, watches, and clocks	Med., Prec., Opt.
123	motor vehicles, trailers, and semitrailers	Mtr., Veh., Trl.
124	other transport equipment	OtherTrans
125	furniture; other manufactured goods n.e.c.^a^	Others

a For details of the classification and the reasoning of modeling inclusion in our model, refer to the “Important Assumptions and Rationales” section in the Supplemental Notes.

#### Input–Output Modeling

We calculated the environmental flows induced by machinery and equipment production using the standard Leontief model^37−39^
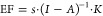
1where EF is the environmental flow driven by the capital flow matrices *K*, *s* refers to the intensities of direct environmental impacts (i.e., GHG emission intensity, material extraction intensity, and metal use intensity in this study), (*I* – *A*)^−1^ is the Leontief inverse matrix, *A* refers to the technical coefficient matrix, *I* is the identity matrix, *K* is the capital flow matrix newly constructed in this study and will be introduced in the consequent section. We use the global multiregional input–output tables from EXIOBASE version 3.8.2^34^ as the EEIOA platform provides high (200) product/services resolution and times series from 1995 to 2015 with the projections made out to 2019. It also covers 44 economy-specific regions and 5 aggregated regions. Furthermore, the EEIOA helps us to obtain the annual environmental footprint (flows). Since the length of the spin-up period is related to the average lifetime of products, dynamic material flow studies on stocks benefit from longer time frames,^22^ which would produce more accurate results. We’ve extended the EEIOA framework back to 1970, given that machinery typically has a lifespan of 10–30 years.^9^ We take the assumption that the interindustry transaction matrices from 1970 to 1994 remain consistent with the average from 1995 to 1997. However, we do not analyze the environmental flows from 1970 to 1994; these years are only considered for the initial setup of the stock.

In subsequent sections, we use the labels “MachFP_Carbon”, “MachFP_Material”, and “MachFP_Metal” to denote the calculated EF values for carbon, material, and metal, respectively. Like “flow” indicators from material flow analysis (MFA), these labels capture annual snapshots.

#### Environmental Extensions

We included greenhouse gas emissions, obtained from EXIOBASE, and material extraction (obtained from EXIOBASE 3 and updated following the new release from materialflows.net hosted by Vienna University of Economics and Business (WU Vienna)),^40^ and IRP global material flows database.^41^ The IRP/WU material extraction data track gross material flows such as ores. Furthermore, given that metals often appear in ores at a lower grade, mingled with numerous nonmetals, we modeled primary metal use^35^ which was obtained from the British Geological Survey.^42^ It represents the actual amount of usable metal extracted from the ore. We allocate material extraction and metal use to sectors following a one-to-one exercise^34^ in the EEIOA extension matrices.

#### Obtaining Capital Flow

To understand the flow of capital, we first consider gross fixed capital formation (GFCF). In line with national economic accounting, GFCF is integrated as a component of the final demand in the EEIOA. Our primary aim was to create a matrix that delineates the producers and users of machinery capital.

(1) Determining GFCF of Machinery Assets. Our initial task was to ascertain the GFCF specific to machinery assets
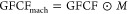
2Here, GFCF [9800*49] denotes the GFCF across 9800 sectors in 49 regions, with GFCF_mach_ [9800*49] illustrating the machinery asset-specific GFCF across these regions (by column). *M* [9800*49] is a selective binary matrix that highlights rows linked to machinery products (as detailed in Table 1). Θ refers to the element-by-element multiplication.
(2) Disaggregation of GFCF Using External Data. We then turned to external data sets to disaggregate the GFCF. These data sets are EU KLEMS 2019^43^ (euklems.eu), World Klems^44^ (worldklems.net), LA Klems^45^ (laklems.net), and national statistics for China,^46^ Norway,^47^ Canada,^36^ and India.^48^ Resolutions on capital asset type and industry classification vary among regions (Table S3). We developed concordance matrices to align these to the 200 product/service resolution of EXIOBASE 3.
(3) Deriving Capital Use Structure. In our analysis, we use the notation “*a*” to represent the aggregated asset type, sourced from external data sets. Subsequently, when we break down this aggregated data into a more detailed resolution to align with the EXIOBASE format, we employ the notation “*p*” to denote the disaggregated asset type aligned with the EXIOBASE product classification. To effectively translate our inputs into the EXIOBASE format, we employed the consumption of fixed capital (CFC) structure from EXIOBASE^6^
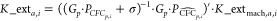
3where *K*_ext_(*a,i*)_ [200, 1] is the machinery capital asset type *a* used in region *i*. It reflects the capital flow structure among sectors in regions *i*. *K_*ext_mach*,a,i*_ [*X*, 1] is the GFCF of machinery assets type *a* (e.g., general machinery, IT, CT, transport equipment, etc.) accumulated in region *i*. Note, *K_*ext_mach*,a,i*_ is not obtained from EXIOBASE but from other sources (such as KLEMS, see above) to derive the splitting ratio of the consuming structure. *G*_*p*_ [*X*, 200] refers to the concordance matrices that transform *X* sectors to 200 product-level consistent with EXIOBASE resolution.  [200, 1] refers to the product-level CFC proxy of region *i*. σ is a small perturbation matrix that ensures nonsingularity. Further details can be obtained from previous works.^4,29^

We aimed to understand the capital use structure of individual regions (*i*). And we need to prepare a ratio matrix for further distribution, Ratio_*K*_*i*_ [200, 200]
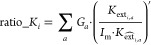
4where *I*_m_ is a summation vector [1*200] and *G*_*a*_ [200, *a*] refers to the concordance matrix to locate the different types of assets *a* into 200 products in EXIOBASE.

Putting all regions together and assuming a region uses domestic and foreign manufactured machinery in a similar manner then we get
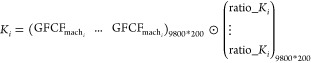
5where GFCF_mach_*i*__ [9800, 1] is the GFCF of machinery assets in region *i* and *K*_*i*_ [9800, 200] refers to the capital flow matrix for region *i*. The column-wise (vertical) elements in *K*_*i*_ indicate the production structure of machinery capital and the row-wise (horizontal) elements in *K*_*i*_ the use structure of machinery capital. Further details can also be found in previous works.^6,29^ In specific regions, data might be missing for certain years. Our strategy to counter this has been to use data from comparable economies to fill these gaps. For instance, for any capital use structural gaps between 1970 and 1994, we utilized an averaged structure from 1995 to 1997. Similarly, for missing data in 2018 and 2019, an average from 2015 to 2017 was taken as a substitute.

Incorporating yield coefficients is challenging. Estimating metal loss during machinery production is difficult, even using methods like the Waste-IO model.^49,50^ Without real-world engineering yield data, assumptions could introduce inaccuracies. As a result, here we do not include the yield coefficients and obtain the full footprint, capturing the entire carbon and material footprints related to machinery production. The stock level reflected by this indicator might be overestimated.

### Calculating Stocks

#### From Flows to Stocks

Using the dynamic material flow analysis method, we assign environmental flows throughout the life cycle of machinery stocks’ life cycle. While investments recorded as GFCF form the addition to stocks, removals can either be modeled using (i) the survival curve, or retirement function, detailing how stock cohorts retire over time,^51,52^ and/or (ii) the depreciation approach, indicating financial accounting’s loss of book value.^29^ This resulting footprint of the stock has also been called the “legacy environmental footprint”, which essentially captures the historic environmental costs from investments forming the current manufactured asset stock.^3^ In our results, we present the machinery stock’s footprint based on the physical stock (survival curve). Subsequently, we contrast the outcomes of the physical stock with its economic value (using depreciation) to discuss the differences and implications.

#### Survival Curve

The lifetime distribution *f*(*x*) is obtained from the literature in the Log-Normal distribution or Weibull distribution.^52^ We considered the difference in the survival curve (lifetime) of different types of machinery and equipment employed in different industries. Such differences are reflected in the specific parameters. The parameters are obtained from.^52−55^*F*(*x*) is the cumulative density function
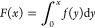
6The survival curve *S(x*) describes the probability that an asset of any vintage survives until age *x* can be written as 1 – *F*(*x*)
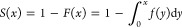
7The stock built in year *m* and remained in year *n* of region *i*, asset *p*, and sector *q* could be presented as
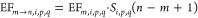
8The total amount of (environmental flows embodied in) stock accumulated in year *n* of region *i*, asset *p*, and sector *q* could be calculated as
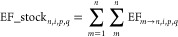
9We represent the calculated accumulated carbon, material, and metal footprint (*EF_stock*) under the labels “MachFP_Carbon of Stock”, “MachFP_Material of Stock”, and “MachFP_Metal of Stock” in the following texts.

#### Depreciation

We use the Perpetual Inventory Method (PIM), a technique widely used in most OECD countries to construct measures of capital stocks for assets.^56^ Further details are noted in a previous work.^29^ In brief, the stock built in year *m* and remained in year *n* of region *i*, asset *p*, and sector *q* could be presented as
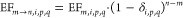
10For the depreciating method, the total amount of (environmental flows embodied in) stock accumulated in year *n* of region *i*, asset *p*, and sector *q* could be calculated as
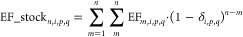
11The depreciating rates are obtained from EU KLEMS 2019^43^ (euklems.eu), World Klems^44^ (worldklems.net), LA Klems^45^ (laklems.net), and the literature.^57,58^ For missing values, we use substitutes from similar economies (Table S3). See Supplemental Notes for additional explanations on measuring elasticity and assumptions and rationales.

## Results

### Growing Carbon and Material Footprints of Machinery

We evaluated the carbon, metal, and material footprints that are derived from machinery production for capital formation and assigned these footprints to the asset users. In the following, these footprints are referred to as MachFP_carbon, MachFP_Material, and MachFP_Metal, respectively. These metrics capture the annual “flow”, providing a snapshot of greenhouse gas emissions, material extraction, and metal use associated with the production of capital goods recorded in the gross fixed capital formation (GFCF) of national economic accounts. To clarify, this measure considers the supply chain factors (impacts created globally) rather than the actual material contained within the machinery assets. This could influence the actual magnitude of the machinery stock given that production efficiencies differ across regions.

**Figure 1:**
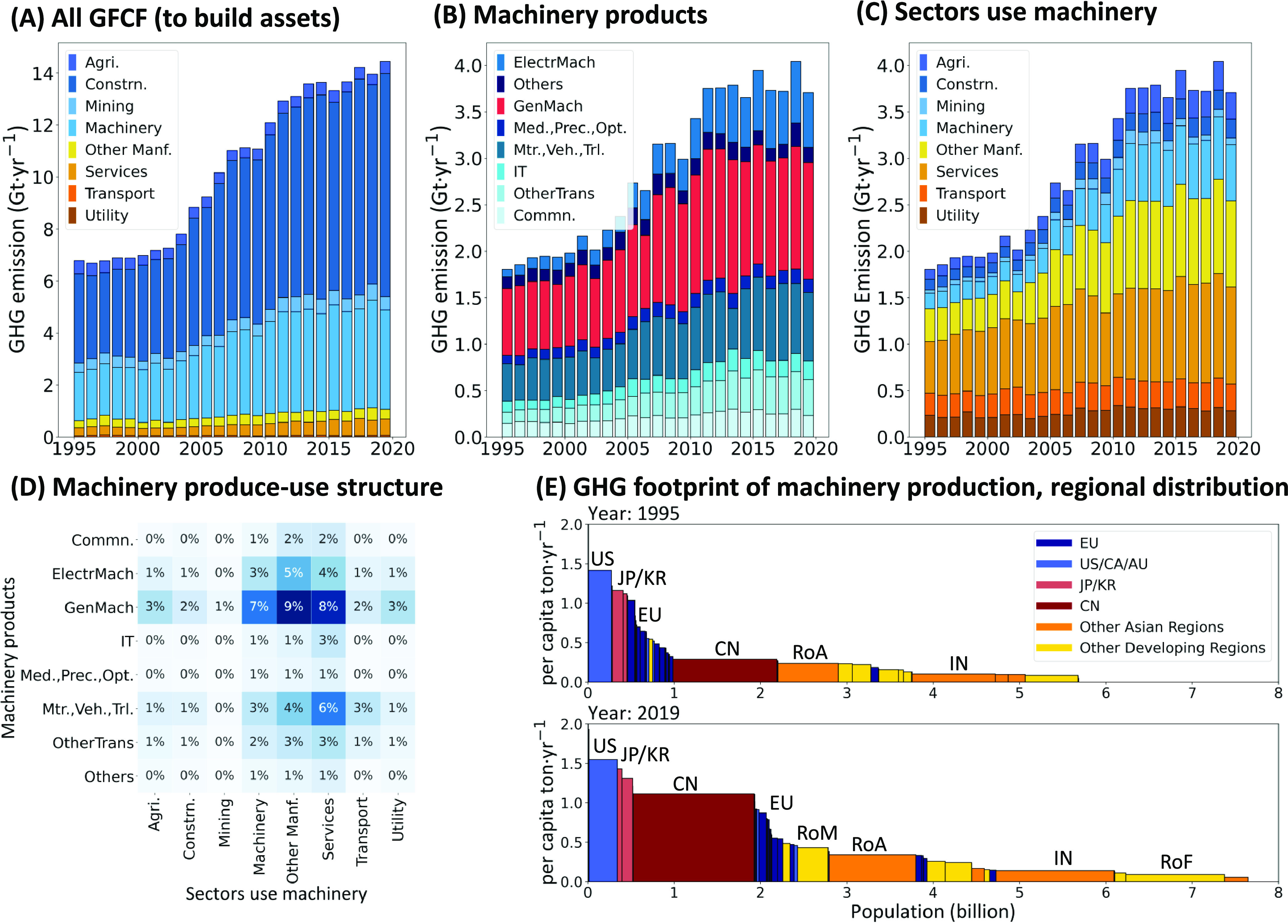
Carbon footprints of machinery production. (A) Carbon footprint driven by Gross Fixed Capital Formation (GFCF): breakdown by product. Machinery parts are depicted in light blue. (B) Carbon footprint of machinery production: breakdown of the “machinery” part in (A) by detailed machinery category. (C) Carbon footprint of machinery production: breakdown of the “machinery” part in (A) by use sector. (D) Use structure of detailed machinery products in 2019 (normalized by the total amount). (E) Carbon footprint of machinery production in a single year of 1995 and 2019. The featured region includes European countries (EU), the United States, Canada and Australia (US/CA/AU), Japan and Korea (JP/KR), China (CN), India (IN), rest of Asian countries (RoA), rest of Middle Eastern countries (RoM), and rest of African countries (RoF). The “Machinery” sector here means the sector (using machinery) to produce machinery products. See Figure S4 for the material footprint and Figure S5 for the metal footprint of machinery production. See Table 1 for the full names of machinery assets.

Machinery and equipment production is a significant contributor to the footprints of GFCF, second only to buildings and infrastructure (Figures 1A, S3, S4A, and S5A). In 2019, they accounted for roughly 26% of carbon, 44% of metal, and 21% of the material footprints of GFCF worldwide (Figure S3). This is approximately 9% of global carbon emissions, 32% of metal usage, and 9% of material consumption (Figure S3). In the United States and Europe, machinery is the primary driver of metal consumption. It accounts for up to 70% of metal demand in capital formation, highlighting machinery as the most crucial repository for carbon-intensive metals.^21^ Among the eight machinery assets, general machinery and equipment, vehicles and transport equipment, and electrical machinery were the top contributors to carbon and materials footprints (Figure 1B). In the economy, manufacturing (mining, machinery production, and other manufacturing) accounts for approximately 40% of machinery asset usage globally, followed by services (30%), utilities (9%), transport (9%), agriculture (7%), and construction (6%) in terms of MachFP_Carbon (Figure 1C,D; refer to Figures S6 and S7 for more details and breakdown by sector, region, and value-added intensity).

The carbon and material footprints resulting from the production of machinery and equipment continued to increase. Between 1995 and 2019, MachFP_Carbon doubled (from 1.9 Gt·yr^–1^ to 3.8 Gt·yr^–1^, Figure 1B,C), while MachFP_Material and MachFP_Metal nearly tripled (from 3.2 to 8.8 Gt·yr^–1^, and 0.2 to 0.7 Gt·yr^–1^, see Figures S4 and S5). Developing countries, particularly China, have driven the growth of machinery production’s footprint (Figure 1E). From 1995 to 2019, China’s rapid economic growth contributed to significant increases in MachFP_Carbon (a 4-fold growth from 0.3 to 1.6 Gt·yr^–1^), MachFP_Material (a 6-fold growth from 0.7 to 4.1 Gt·yr^–1^), and MachFP_Metal (a 14-fold growth from 0.02 to 0.3 Gt·yr^–1^). For reference, China’s GDP increased 8-fold over the same period. Other developing countries such as India, Russia, and Turkey also had a 3-fold growth in their footprints. In contrast, the footprints of machinery grew slowly and flattened out in advanced economies.

In terms of per capita measurements, developed countries still have higher machinery-related footprints. For example, carbon footprints grew in the United States (growing from 1.4 to 1.6 t/cap during 1995–2019), Korea (from 1.1 to 1.5 t/cap), and Japan (from 1.2 to 1.3 t/cap). China is narrowing the gap with these developed countries, escalating from 0.3 t/cap (19% of the US) to 1.1 t/cap (approximately 70% of the US). Other developing economies are approaching the per capita carbon footprints of developed nations. For instance, in 2019, the machinery carbon footprint (MachFP_carbon) of Turkey (0.5t/cap), Middle Eastern Countries (RoM) (∼0.4t/cap), and Asian Countries (RoA) (∼0.3t/cap) matched or surpassed levels seen in the United Kingdom (∼0.3t/cap) and Eastern European countries (∼0.3t/cap).

### Stock Expansion and Geographical Shifts

The accumulated capital goods form the capital stock that functions to provide services to the economy. By using the survival curve approach, we define the terms “MachFP_Carbon of Stock”, “MachFP_Material of Stock”, and “MachFP_Metal of Stock” to represent the legacy carbon, material, and metal footprints^3^ of machinery stocks. Without direct cohort data, these indicators do not reflect the exact physical stock size but offer reasonable proxies of in-service machinery cohorts across regions.

**Figure 2:**
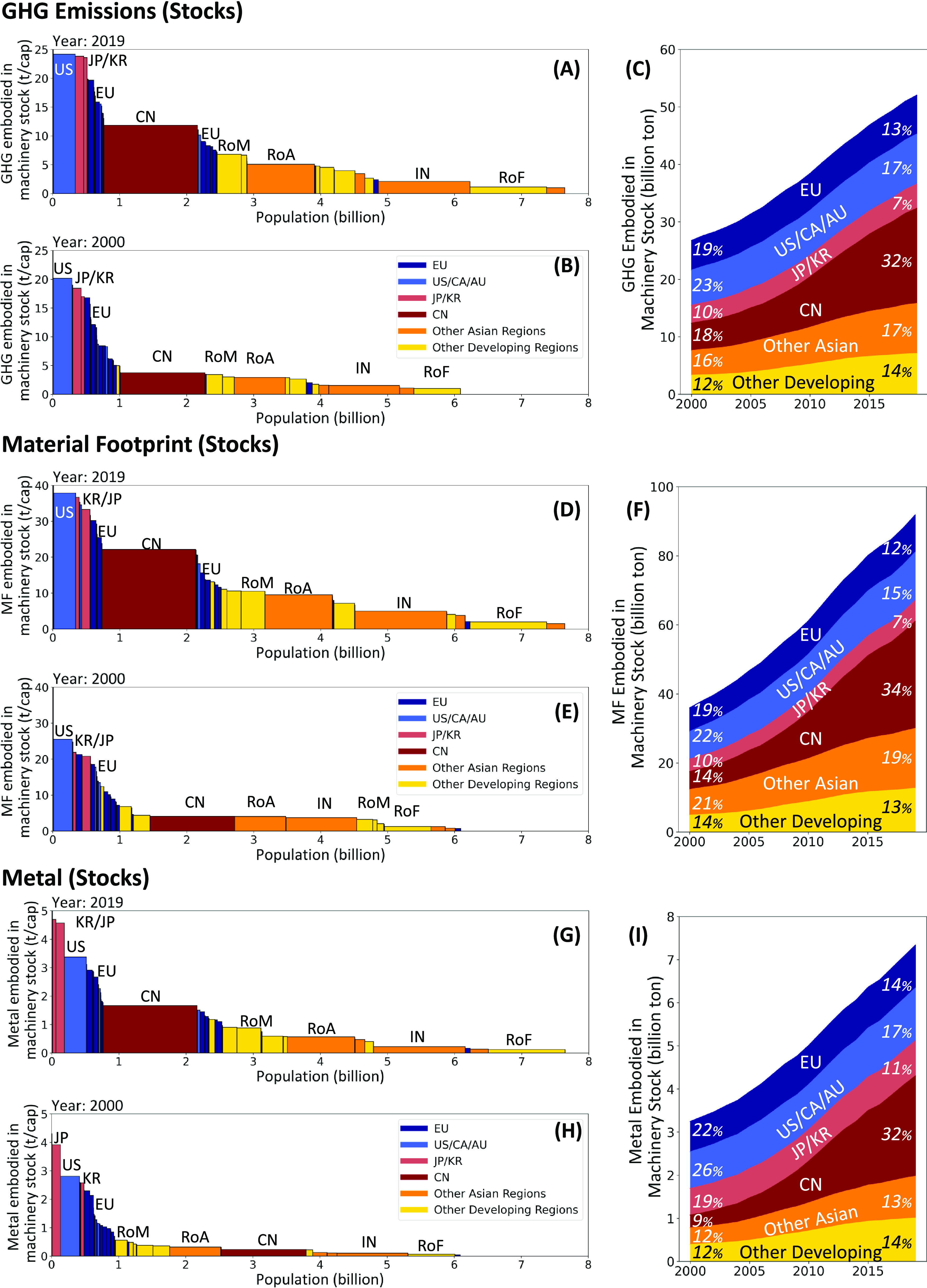
Regional comparison of carbon, material, and metal footprints in machinery and equipment capital stock. Analysis of MachFP_Carbon, MachFP_Material, and MachFP_Metal in 2019 (A–G) and 2000 (B, E, H). Growth of MachFP_Carbon (C), MachFP_Material (F), and MachFP_Metal (I) embodied in global machinery stocks. The featured region includes European countries (EU), the United States, Canada and Australia (US/CA/AU), Japan and Korea (JP/KR), China (CN), India (IN), rest of Asian countries (RoA), rest of Middle Eastern countries (RoM), and rest of African countries (RoF).

The results demonstrate a doubling of the legacy environmental footprint of machinery stock during the first 20 years of this century (Figure 2). Between 2000 and 2019, MachFP_Material of Stock grew from 35.9 to 91.8 Gt (5.9 to 12.0 t/cap), MachFP_Metal of Stock from 3.3 to 7.3 Gt (0.5 to 1.0 t/cap), and MachFP_Carbon of Stock from 26.7 to 52.0 Gt (4.4 to 6.8 t/cap) globally. In 2019, the countries with the largest MachFP_Metal of Stock were China (2.3 Gt), the US (1.1 Gt), Japan (0.6 Gt), India (0.3 Gt), Korea (0.2 Gt), and Germany (0.2 Gt). In 2000, China’s MachFP_Metal of Stock was only 37% of that of the US. However, China’s MachFP_Metal of Stock surpassed the US’s around 2009 (Figure S8) and was twice as large in 2019 (China: 2.3 Gt; US: 1.1 Gt). Similar trends are observed for MachFP_Carbon of Stock (China: 16.5 Gt; US: 7.9 Gt) and MachFP_Material of Stock (China: 30.9 Gt; US: 12.4 Gt) in 2019.

The remarkable growth in developing regions reflects a shift in the global production system. The highest growth rate of per capita MachFP_Metal of Stock occurred in developing regions such as China (an 8-fold increase), Russia (4.5-fold), Middle Eastern regions (3.5-fold), and Turkey (3.3-fold), while other Asian countries lagged behind (2.4-fold). Noticeable increases were also observed in certain advanced European countries (e.g., Ireland) and emerging Eastern European countries. However, overall, the growth rate and absolute increase in developing regions far exceeded that of developed countries. As a result, the advanced economies’ proportion of the global MachFP_Material of the stock decreased from 51 to 34% (as shown in Figures S9–S11). The change was even more significant for machinery used in production industries (extraction, manufacturing, and machinery), where in 2019, advanced economies only accounted for 24% (down from 45%) of the worldwide share.

The per capita MachFP_Metal of Stock in advanced economies was still high. Switzerland exhibited the highest per capita MachFP_Metal of Stock (5.0 t/cap) in 2019 (see a further discussion in the SI), followed by Korea (4.7 t/cap), Japan (4.6 t/cap), and the US (3.4 t/cap). In 2019, the per capita MachFP_Metal of Stock in China (1.7 t/cap) was only half that of the US. India’s MachFP_Carbon of Stock slightly exceeded that of Germany, but its per capita value was only 4% of Germany’s. From 2000 to 2019, not all developing economies experienced substantial growth in machinery stocks. Moderate growth (below the global average: +80%) was observed in Indonesia (+8%), South Africa (+36%), and Brazil (+61%), which can be partially attributed to underinvestment in industrial systems and high population growth.^59^ The observed low per capita saturation in some developing countries (in Africa and South America) is noteworthy. This may imply a potential stagnation in their participation in industrial value chains.

In developed countries, the distribution of the per capita stocks was uneven. Switzerland’s MachFP_Metal of Stock (5.0 t/cap) was three times the European average (1.7 t/cap). Western European countries, such as Belgium and Germany (both around 2.9 t/cap), generally surpassed Eastern European countries (<1.5 t/cap). Furthermore, limited growth or even declines were observed in the UK and Poland, both of which maintained MachFP_Metal of Stock around 1 t/cap throughout the years, reflecting a process of deindustrialization.

**Figure 3:**
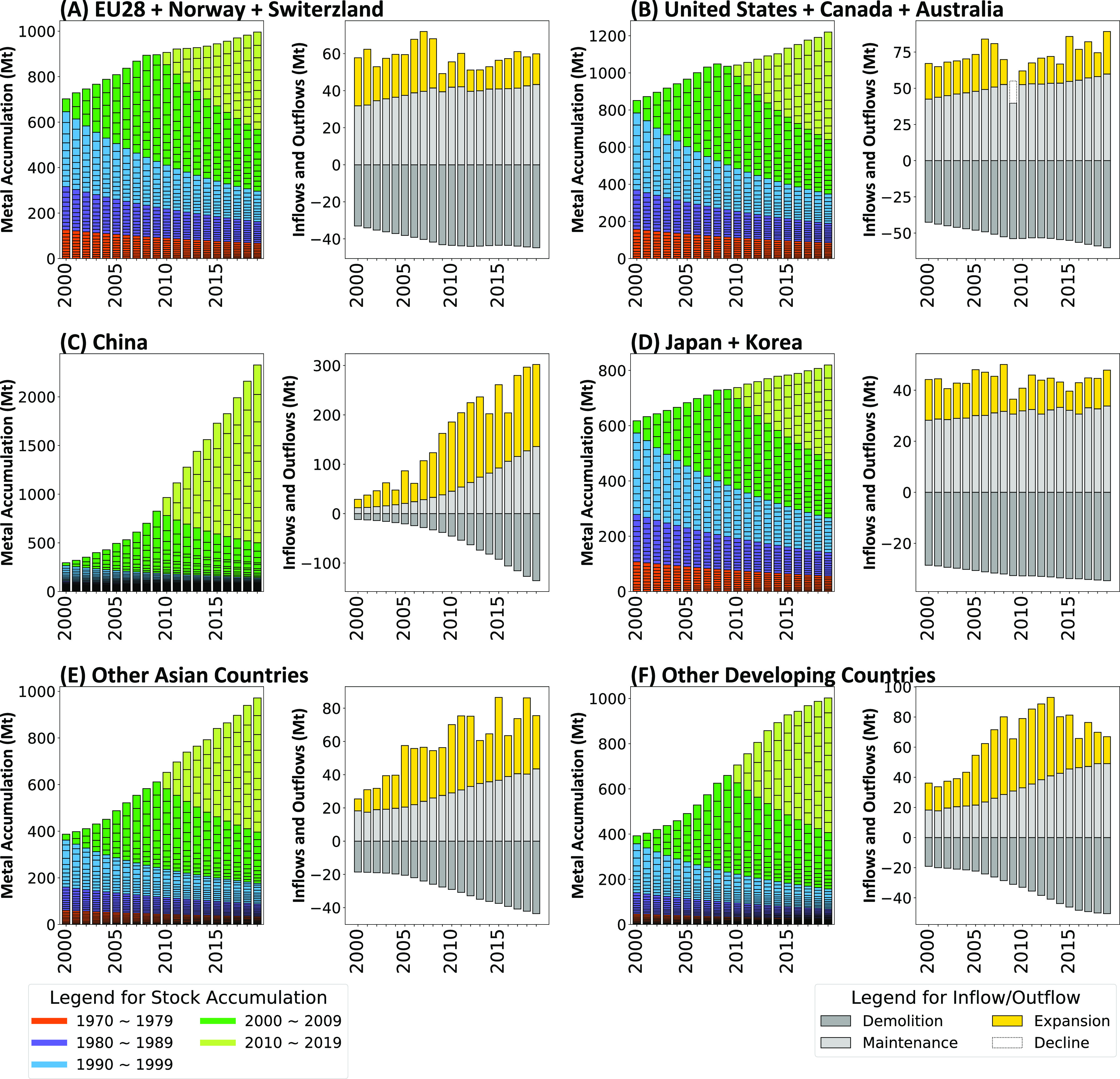
Metal footprint embodied in machinery stock and inflows, and outflows across different regions. (A–F) Different region groups (see Supplementary Notes for grouping classification). The first and third columns indicate the metal footprint embodied in machinery stocks over the years, while the second and fourth columns indicate the inflows and outflows that include demolition, maintenance, expansion, and decline. For detailed definitions, see Figure S13. See Figures S14 and S15 for the results of the carbon footprint and the material footprint analysis. Results were calculated using the survival curve. See Figures S16–S18 for the results derived through depreciation. See Table S1 for the regional grouping.

Differentiating the inflows (for maintenance and expansion) and outflows (demolition) of MachFP_Carbon of Stock further elucidates the capital dynamics underlying the geographic shifts (Figure 3). We define maintenance (including replacement) here as the annual inflows necessary to make up for the annual outflows during a capital expansion (see Figure S13 for details). Over the past decade, maintenance constituted 80% or more of the investment in developed economies. In developing regions such as China, Asia, and others, meanwhile, about half of the inflows contributed to machinery stock expansion (Figure 3). This is consistent with our findings that the machinery stock in developed regions is relatively old, resulting in an ongoing replacement; hence, the demolished stock itself can become the source of metals to produce its replacement, ensuring a circular economy. By contrast, the stock in developing areas is relatively young. Given its recent expansion, it will be imperative to establish a corresponding circulation system in the future.

### Differences between Physical Accounts and Financial Values

Several studies have noted that in affluent countries, iron and steel stocks grew for a time before plateauing around 10–12 t/cap.^60^ This seems to suggest that new investments replaced existing stocks rather than added to them. The same saturation trend has been inferred for other metals.^9,61−64^ Consequently, we must ask: Could a similar trend be observed in the material and carbon footprints of machinery assets?

Our data indicate continuous growth in the machinery stock indicators (MachFP_carbon, MachFP_Material, and MachFP_Metal of Stock) across most regions (Figure 3). Some sectors and regions, such as the extraction sector in developed areas (Figure S19), Machinery Manufacturing in the US and Japan (Figures S20 and S21), and Japan’s construction domain, exhibit signs of potential saturation and decline in terms of MachFP_Metal of Stock. However, on a broader scale, the machinery capital footprint does not seem to be hitting saturation. Both regional and global machinery stocks continue to grow, evident in both per capita and absolute metrics. An exception to this trend is the noticeable stagnation in per capita metrics in certain low-income countries.

In finance and national economic accounts, capital stock quantification, primarily based on depreciation, focuses on the financial value rather than the tangible or productive value of materials. Machinery serves dual roles: it aids in efficient economic value creation and holds the potential for resource recovery post use. The economic value of assets, reflected by geometric discounting,^65^ declines exponentially, while their resource value stays stable until they’re recycled or discarded. Environmental analysis has adopted both these perspectives.^29,66^ While footprint studies trace the environmental impacts of capital production based on fixed capital consumption and depreciation^4,5,29^ others emphasize the actual physical quantity of materials, using survival rate functions to represent material stock lifespan.^51,67^

**Figure 4:**
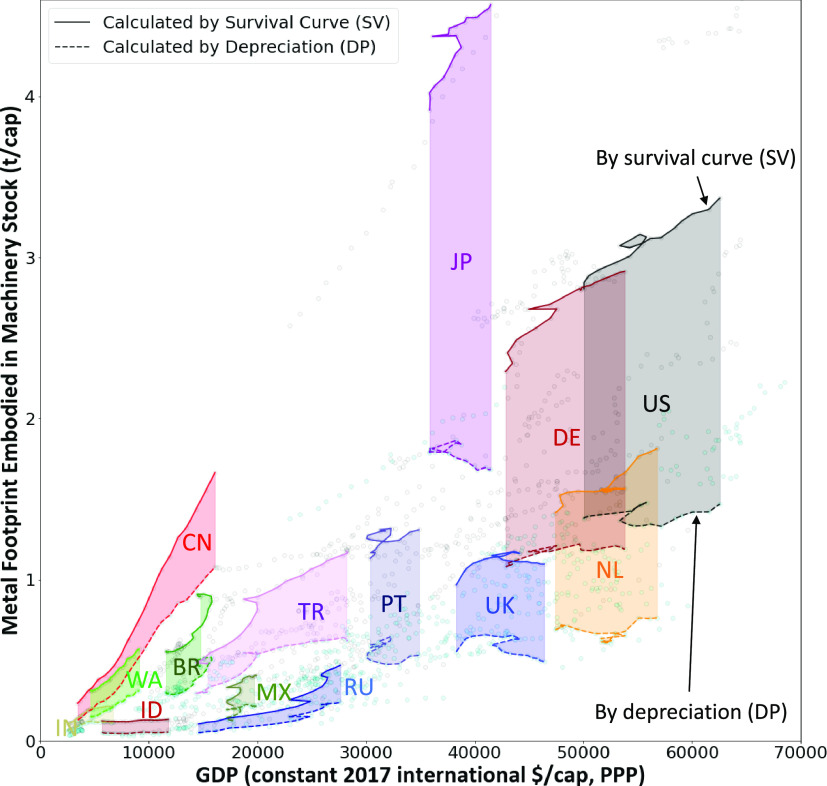
Metal footprint embodied in machinery stock: Comparison of physical world vs financial present values. The metal footprint is calculated by using the survival curve (SV) and depreciating rates (DP) for different countries. Solid lines depict the results based on the survival curve, while the dotted lines below indicate outcomes derived from depreciation rates. Note, we need to be careful in interpreting the capital stock footprint; we use them as proxies of the sizes of capital stocks, but they actually measure the emissions, materials, etc. historically took them to build them. The *X*-axis represents the GDP at purchasing power parity (PPP, constant 2017 international dollars) per capita for each economy. The shaded area highlights the discrepancy between metals contained in tangible assets and those linked to the present asset values. Featured countries include Brazil (BR), China (CN), Germany (DE), Indonesia (ID), India (IN), Japan (JP), Mexico (MX), Netherlands (NL), Portugal (PT), Russia (RU), Turkey (TR), United Kingdom (UK), United States (US), and the Rest of Africa (WA).

In our study, as shown in Figure 4, we assess the machinery stock footprint using both the survival function (SV in the upper solid lines) and the depreciation curve (DP in the dashed lines below). The gap between physical accounts and financial values goes beyond the mere measurement differences. It fundamentally highlights the contrast in our real-world valuation of assets with their paper accounting.

First, in advanced economies like Japan, the U.S., and Germany, the difference between the survival function (SV) and depreciation rate (DP) is pronounced, with SVs exceeding DPs by two to three times. This difference is due to the unique patterns of both curves (see Figure S22). For example, while vehicles are expected to lose half of their value by the fifth year, most are still in use beyond that.

Second, as wealth increases, the difference between the survival function (SV) and depreciation rate (DP) grows. In emerging countries such as China and Turkey, the SV-DP gap is smaller than in developed ones. Initially, during capital accumulation phases, both SV and DP curves rise at similar rates due to significant capital formation. However, over time, this gap will likely widen, resembling patterns in advanced nations.

In developed countries, while DP curves show signs of stagnation or decline, SV curves continue to rise. This disparity in our findings warrants further investigation. Financial reporting, adhering to traditional depreciation standards, frequently influences investment choices. These financial choices often result in the formation of tangible assets. This raises the following question: do these pronounced discrepancies suggest diverse interpretations of the saturation challenge in various studies? If these curves accurately capture the present situation, they reveal a significant volume of capital invested that, while depreciating, remains functional. Even though this capital has zero book value, it still plays a vital role in production, service delivery, and associated energy consumption.

The gap between tangible asset values and their economic evaluations has profound implications for both financial and environmental strategies. The distinct patterns observed in depreciation and survival functions can result in varying assessments of a capital’s environmental impact. This discrepancy might lead companies to either discard assets too early, wasting potential utility, or neglect the operational value of assets that have seen financial depreciation. Financial reporting that may overemphasize new assets rather than maintain existing ones (as they have limited monetary values on the books) may cause firms to overlook potential sustainability and cost-saving benefits. The identified inconsistencies point to a need for refined asset valuation models that more accurately represent tangible and functional values, especially in industries with long-term assets. To be specific, when the environmental effects of capital are evaluated, the chosen methodology is crucial. For instance, decisions in corporate strategies for Scope 3 emissions reporting can greatly affect the allocation of emissions linked to capital, leading to significant variations in reported figures and reshaping the distribution of responsibility.

## Discussion

In recent decades, machinery and equipment have risen as a significant contributor to global carbon, metal, and material footprints, highlighting their crucial environmental impact. Their influences are only surpassed by those of the buildings and infrastructure sectors. The footprint of the capital stock primarily measures the historical emissions and materials required for their production. It is important to emphasize that the capital stock footprint encompasses not only the materials within the capital stock but its entire supply chain as well: the footprint efficiency of the production system in addition to the size of the stock. Consequently, due to improved energy efficiency and a cleaner energy supply, the carbon footprint of machinery stock grows at a more moderated rate than its metal and material counterparts (Figure S23).

Building on this, we reviewed previous studies^68−75^ and found consistent outcomes (Table S4). One aspect not factored in is the effect of yield: the volume of materials retained in the product during its production. While potential yield calculations could stem from studies like Waste-IO,^49,50^ existing work lacks empirical data on yield coefficients from the industry. Alternative studies, especially those addressing specific waste flows, may be beneficial in deducing detailed yield coefficients. Still, obtaining comprehensive data presents a challenge. Future research would benefit from bottom-up studies that collect physical data.

We explored the relationship between value creation and machinery stock across diverse sectors (see SI for results and methodological specifics). Notably, higher elasticity (>∼1) is witnessed in the transportation, utility, and service sectors (Table S2). These sectors, primarily asset-light, have an edge in leveraging machinery to bolster output effectively.^76^ Their operational flexibility further amplified this, fostering swift machinery or technology integration, thereby elevating service quality and value. In contrast, sectors like mining and agriculture demonstrate lower elasticity (<0.5). Here, surges in machinery do not correlate to considerable value boosts, potentially due to an overreliance on other resources or machinery underutilization. Manufacturing presents an elasticity around 0.8, with GHG standing out at 1.1. Such sectorial elasticity insights could provide references for integrated assessment modeling.

The significant influence of machinery on sustainable development deserves crucial attention, as its long-term operational impacts on the environment are profound.^4−6,77,78^ In China, key machinery and equipment (boilers, motors, power transformers, refrigeration, lighting, household appliances, etc.) operation accounts for about 80% of total energy consumption.^79^ This shows promising opportunities to save energy and cut down on carbon emissions. With developed regions exhibiting extended machinery lifetimes^9^ and China’s rapid machinery growth nearing its plateau, the country has implemented policies aimed at energy savings and resource recovery.^79^ This brings up a question: should we replace old machines with more efficient ones or use what we already have to save on materials? Either way, using methods that reuse and recycle, like remanufacturing, can help a lot in cutting carbon emissions and preserving metals.^80^

In the pursuit of a climate-neutral society, the implications of machinery production warrant significant attention, given their dual role: they serve as vital technological carriers for transition, especially in emerging economies, yet they also pose environmental challenges. It also becomes important to revisit the valuation frameworks for these assets, integrating both financial and environmental perspectives. Future analysis including scenario-based modeling will better identify circular economy and climate mitigation options for society’s most considerable use of metals and develop better demand scenarios for materials.

## Supporting Information Available

Expanded description of the methodology, important assumptions, underlying rationales, and data sources; additional results including elasticity measures, supplementary figures, and tables; extended discussion on the per capita footprint of machinery stocks (PDF)
Supplementary Data set: the results of “MachFP_Carbon of Stock”, “MachFP_Material of Stock”, and “MachFP_Metal of Stock”, along with their respective breakdowns; the results used for regression analysis (XLSX)
