# Supplementary material for: Material and Carbon Footprints of Machinery Capital
Source: Environ Sci Technol. 2023 Nov 22;57(50):21124–35. doi: 10.1021/acs.est.3c06180 (PMC10734266; doi:10.1021/acs.est.3c06180)
Supplement: Supplementary file 1 — es3c06180_si_001.pdf [file es3c06180_si_001.pdf]

Supporting Information for

Material and Carbon Footprints of Machinery Capital

Meng Jiang[1,*], Ranran Wang[2], Richard Wood[1], Kajwan Rasul[1], Bing Zhu[3], Edgar Hertwich[1]

1. Department of Energy and Process Engineering, Norwegian University of Science and Technology, Trondheim 7491, Norway
2. Institute of Environmental Sciences (CML), Leiden University, Einsteinweg 2, 2333 CC Leiden, The Netherlands
3. Department of Chemical Engineering, Tsinghua University, Beijing 100084, China

*Correspondence: meng.jiang@ntnu.no (M.J.)

Supplemental Notes. Figures S1–S24. Table S1-4. 33 Pages.

Contents

Supplemental Notes.. 2
Measuring Elasticity.. 2
Important Assumptions and Rationales.. 2
Regional Alternatives.. 2
GFCF and Asset.. 2
Inclusion of "Furniture; other manufactured goods n.e.c." into "Machinery and Equipment" product sector.. 3
Further discussion on high per capital footprint of machinery stock of Switzerland........................ 3
Data availability.. 3
Supplemental Figures 4
Supplemental Tables.. 23
Reference 32

Supplemental Notes

Measuring Elasticity

We used a fixed effects regression model indicated in Equation 1 to map the elasticity of value added (by use-sector, deflated) relative to the machinery stock level (expressed in terms of carbon, metal, and material footprint embodied in the machinery stock). The regression equations are as follows:

$$\log(VA_q) = \beta_0 + \beta_1 * \log\left({Mach_{FP}}_q\right) + u_i + \epsilon \qquad (1)$$

Here VA_q denotes the value added of sector q. ${Mach_{FP}}_q$ represents the footprint embodied in the same sector q. β_0 is an individual invariant effect (intercept). β_1 characterizes the relative change (elasticity) in VA_q to ${Mach_{FP}}_q$. It indicates that a 1% increase in ${Mach_{FP}}_q$ is associated with an average change of β_1% in VA_q. u_i is the regional fixed effects and ϵ is the error term. The results of this regression model are presented in Table S2.

Important Assumptions and Rationales

Regional Alternatives

We collected parameters for the survival curve [1-4] for machinery assets, classified by their manufacturing category and usage sector as best as we could. However, due to the unavailability of region-specific data, we had to assume that the equipment survival curves for each region are identical.

Upon consulting industry experts, we found that there might be minor variations in life expectancy among countries, though these differences are not significant. For construction machinery specifically, there is a limited number of leading manufacturers, and their products can be considered analogous. Maintenance and servicing are usually handled by professionals, which further reduces any potential gaps. A possible difference could be that equipment is used more intensively in developing regions, such as China, which in turn leads to a shorter lifespan. Nonetheless, since equipment in developing countries' cohorts is relatively young, it does not impact the results considerably.

In cases where capital flow statistics and depreciation data were unavailable due to data scarcity, we employed structures from analogous regions. Refer to Table S3 for more information.

GFCF and Asset

In practice, the GFCF of machinery production-related industries does not equate to the actual machinery physical asset size (book value). This discrepancy arises from the effects of yields, machinery-related services, and intangible assets. Making rough assumptions is not beneficial, as production rates across industries are not accessible. Consequently, we used metal, material, and carbon footprint as proxies to estimate a region's machinery stock level. Future research utilizing an independent estimate of capital stock, gauged, for example, by production capacity or capital value, would provide increased accuracy.

Furthermore, we compared GFCF values for these assets (sectors/products) from PWT (https://www.rug.nl/ggdc/productivity/pwt/?lang=en), Klems (https://euklems.eu/), and EXIOBASE. Although they show similarities, occasional discrepancies exist. To ensure consistent results from the GMRIO model when allocating environmental impacts, we employ the total GFCF data from EXIOBASE as the benchmark and apply the structure from other data sources for splitting purposes. Physical-based indicators, unaffected by price changes, adhere to the principle of mass conservation.

Inclusion of "Furniture; other manufactured goods n.e.c." into "Machinery and Equipment" product sector

Our decision to include the product sector in EXIOBASE "Furniture; other manufactured goods n.e.c." stemmed from our reference to Statistics Canada (https://www150.statcan.gc.ca/t1/tbl1/en/tv.action?pid=3610009701). They categorize Furniture and related products, as well as Other manufactured products and custom work, under the umbrella of Machinery and equipment. This particular classification encompasses manufactured goods that aren't distinctly categorized, inclusive of certain specific machinery and equipment. We hence follow this classification to be more comprehensive.

The "other manufactured goods n.e.c." is a catch-all category for items not classified elsewhere, some of which could be machinery or equipment.

Furniture, especially when considering items like office chairs or specialized workstations, can be seen as equipment. Machinery and furniture, when understood in a narrower context, frequently share common manufacturing processes, including metalworking, carpentry, welding, assembly, and finishing. Both fall under the category of capital goods, implying they aren't directly consumed but play a pivotal role in facilitating the production of other commodities. Notably, both industrial machinery and commercial-grade furniture are characterized by their longevity, representing enduring investments that undergo depreciation throughout their lifespan. Such assets also necessitate consistent maintenance and care. They facilitate certain tasks and activities.

In addition, as depicted in Figure 1B and 1D, the contribution of "Furniture; other manufactured goods n.e.c." is relatively tiny, thereby minimally influencing the overarching trend of our results of the “core” machinery and equipment.

Further discussion on high per capital footprint of machinery stock of Switzerland

The pronounced values for Switzerland in comparison to Germany can be largely attributed to a combination of potential factors. Switzerland's smaller population can amplify per capita metrics (such as the manufacturing value added per capita, https://w3.unece.org/SDG/en/Indicator?id=130). Its manufacturing sector is particularly strong in high-value domains like precision instruments and pharmaceuticals. And further, Switzerland's inclination to import machinery and equipment, which might possess a higher footprint than domestically produced items, could also be a contributing factor. This is an issue worthy of further exploration, and subsequent bottom-up analysis will be important.

Data availability

The capital-consuming data and depreciating rates are obtained from EU KLEMS 2019 [5] (https://euklems.eu/), World Klems (https://www.worldklems.net/), LA Klems (http://laklems.net/), and national statistics for China[6], Norway[7], Canada[8], India[9], and literature[10, 11]. The parameters of the survival curve could be obtained from [1-4]. EXIOBASE could be obtained (https://zenodo.org/record/5589597) and the updated material extraction data are hosted in (https://www.materialflows.net/) and IRP global material flows database (https://www.resourcepanel.org/global-material-flows-database). Metal content extensions are obtained from British Geological Survey (https://www2.bgs.ac.uk/mineralsuk/statistics/wms.cfc?method=searchWMS). Socioeconomic data including GDP in constant price, GDP-Purchasing power parity (PPP), and deflator are all obtained from World Bank (https://data.worldbank.org/indicator). Results generated in this study can be found in the supplemental dataset.

Supplemental Figures

Consumption	CN	EU	JP_KR	OtherAsia	OtherDevelop	US_CA_AU	Grand Total
Electrical machinery and apparatus n.e.c. (31)	21%	28%	15%	30%	20%	44%	23%
Furniture; other manufactured goods n.e.c. (36)	77%	74%	35%	65%	63%	73%	68%
Machinery and equipment n.e.c. (29)	0%	4%	7%	11%	13%	13%	6%
Medical, precision and optical instruments, watches and clocks (33)	2%	41%	19%	33%	53%	22%	33%
Motor vehicles, trailers and semi-trailers (34)	25%	48%	45%	42%	61%	34%	37%
Office machinery and computers (30)	18%	43%	19%	35%	24%	22%	25%
Other transport equipment (35)	4%	33%	5%	25%	32%	14%	12%
Radio, television and communication equipment and apparatus (32)	75%	64%	62%	47%	49%	41%	50%
Grand Total	12%	33%	17%	29%	35%	30%	22%

GFCF	CN	EU	JP_KR	OtherAsia	OtherDevelop	US_CA_AU	Grand Total
Electrical machinery and apparatus n.e.c. (31)	79%	72%	85%	70%	80%	56%	77%
Furniture; other manufactured goods n.e.c. (36)	23%	26%	65%	35%	37%	27%	32%
Machinery and equipment n.e.c. (29)	100%	96%	93%	89%	87%	87%	94%
Medical, precision and optical instruments, watches and clocks (33)	98%	59%	81%	67%	47%	78%	67%
Motor vehicles, trailers and semi-trailers (34)	75%	52%	55%	58%	39%	66%	63%
Office machinery and computers (30)	82%	57%	81%	65%	76%	78%	75%
Other transport equipment (35)	96%	67%	95%	75%	68%	86%	88%
Radio, television and communication equipment and apparatus (32)	25%	36%	38%	53%	51%	59%	50%
Grand Total	88%	67%	83%	71%	65%	70%	78%

Figure S1 Metal Footprint: Breakdown by Consumption (Household & Government) and Gross Fixed Capital Formation (GFCF) in 2019. The percentage values represent the proportion of the metal footprint attributed to consumption or GFCF in relation to the total metal footprint. The footprint driven by consumption and GFCF (including inventories) sum to the total footprint of a region (i.e., there is no residual footprint).

Material Footprint of Consumption (Household & Gov Purchases): Focus on Machinery and Equipment (2019)

	CN	EU	JP_KR	OtherAsia	OtherDevelop	US_CA_AU
**Electrical machinery and apparatus n.e.c. (31)**	46%	7%	8%	11%	5%	9%
**Furniture; other manufactured goods n.e.c. (36)**	2%	32%	10%	22%	29%	39%
**Machinery and equipment n.e.c. (29)**	1%	2%	10%	7%	9%	7%
**Medical, precision and optical instruments, watches and clocks (33)**	0%	7%	4%	5%	6%	3%
**Motor vehicles, trailers and semi-trailers (34)**	40%	25%	33%	13%	33%	26%
**Office machinery and computers (30)**	6%	6%	6%	4%	3%	4%
**Other transport equipment (35)**	4%	7%	2%	4%	5%	3%
**Radio, television and communication equipment and apparatus (32)**	3%	13%	27%	35%	10%	10%
**Total**	**100%**	**100%**	**100%**	**100%**	**100%**	**100%**

Carbon Footprint of Consumption (Household & Gov Purchases): Focus on Machinery and Equipment (2019)

	CN	EU	JP_KR	OtherAsia	OtherDevelop	US_CA_AU
**Electrical machinery and apparatus n.e.c. (31)**	39%	7%	6%	10%	4%	7%
**Furniture; other manufactured goods n.e.c. (36)**	2%	31%	11%	21%	29%	38%
**Machinery and equipment n.e.c. (29)**	1%	3%	11%	7%	10%	8%
**Medical, precision and optical instruments, watches and clocks (33)**	0%	7%	4%	5%	5%	3%
**Motor vehicles, trailers and semi-trailers (34)**	45%	26%	37%	15%	35%	28%
**Office machinery and computers (30)**	6%	6%	5%	4%	3%	4%
**Other transport equipment (35)**	4%	7%	2%	3%	4%	2%
**Radio, television and communication equipment and apparatus (32)**	3%	13%	25%	35%	10%	10%
**Total**	**100%**	**100%**	**100%**	**100%**	**100%**	**100%**

Metal Footprint of Consumption (Household & Gov Purchases): Focus on Machinery and Equipment (2019)

	CN	EU	JP_KR	OtherAsia	OtherDevelop	US_CA_AU
**Electrical machinery and apparatus n.e.c. (31)**	46%	9%	7%	15%	5%	10%
**Furniture; other manufactured goods n.e.c. (36)**	1%	17%	5%	11%	11%	22%
**Machinery and equipment n.e.c. (29)**	1%	4%	20%	14%	15%	14%
**Medical, precision and optical instruments, watches and clocks (33)**	0%	5%	3%	3%	6%	2%
**Motor vehicles, trailers and semi-trailers (34)**	44%	42%	45%	25%	49%	40%
**Office machinery and computers (30)**	3%	3%	3%	3%	2%	3%
**Other transport equipment (35)**	5%	11%	4%	4%	6%	3%
**Radio, television and communication equipment and apparatus (32)**	1%	9%	13%	26%	6%	6%
**Total**	**100%**	**100%**	**100%**	**100%**	**100%**	**100%**

Figure S2 Footprint of Household & Government Purchases: Focus on Machinery and Equipment. The data is for 2019. The percentage represents the proportion of related categories in a country.

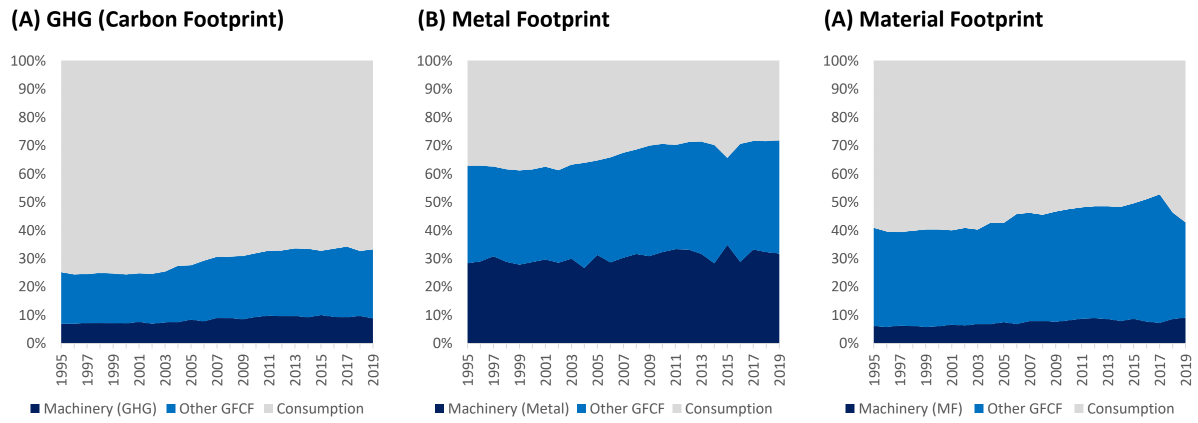

Figure S3. Environmental Footprint of Machinery Production (in Navy), Gross Fixed Capital Formation (GFCF) (in Blue), and Total Footprint: (A) Greenhouse Emissions (Carbon Footprint); (B) Metal Footprint; (C) Material Footprint. The figures show the environmental footprint breakdown of machinery production (as a part of GFCF) and GFCF (excluding machinery), with an emphasis on carbon, metal, and material footprints. Additionally, the contribution to the overall footprint from consumption (including household expenditure and government spending) is presented in grey.

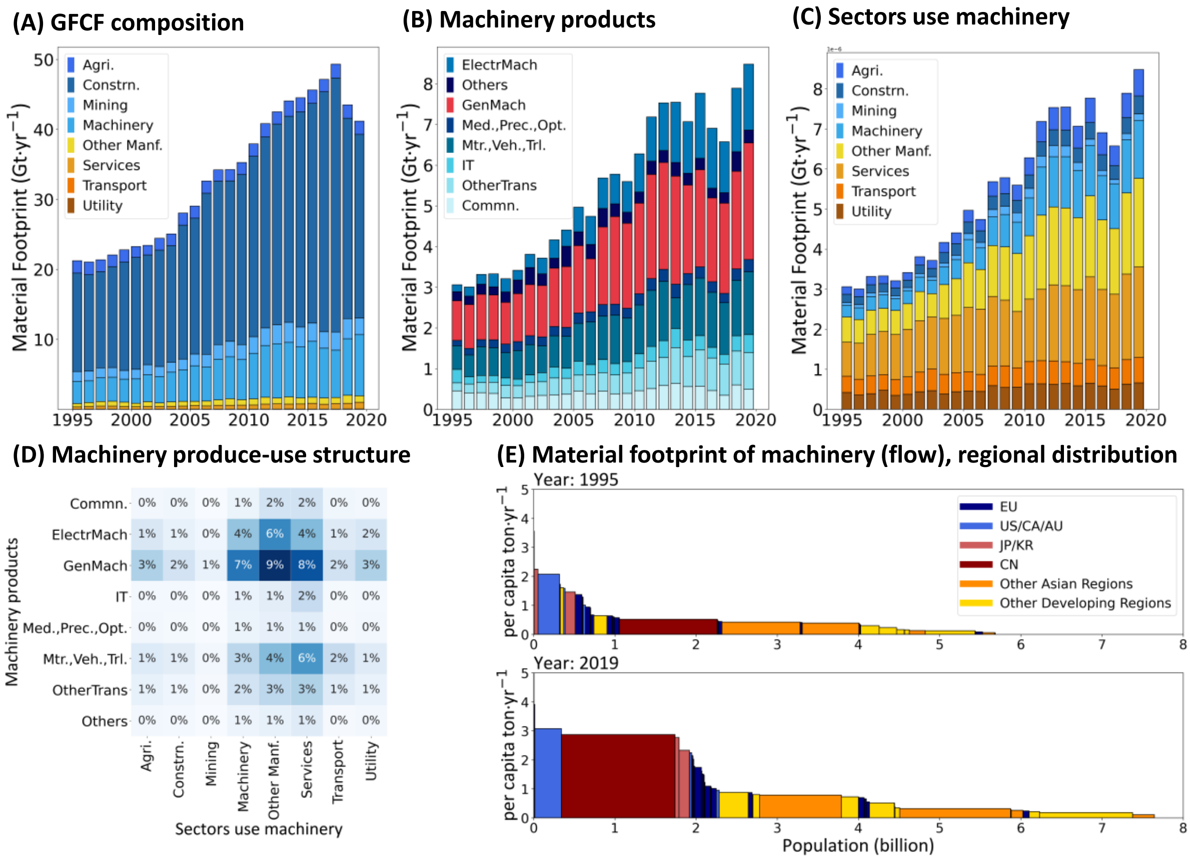

Figure S4 Material Footprints of Machinery Production. (A) Material footprint driven by Gross Fixed Capital Formation (GFCF): breakdown by product. Machinery parts are depicted in light-blue. (B) Material footprint of machinery production: breakdown by detailed machinery category. (C) Material footprint of machinery production: breakdown by use sector. (D) Use structure of detailed machinery products in 2019 (normalized by the total amount). (E) Material footprint of machinery production in 1995 and 2019. The figures show the material footprint of machinery production, broken down by product, category, use sector, and region. The featured region includes European countries (EU), the United States, Canada and Australia (US/CA/AU), Japan and Korea (JP/KR), China (CN), India (IN), rest of Asian countries (RoA), rest of Middle Eastern countries (RoM) and rest of African countries (RoF).

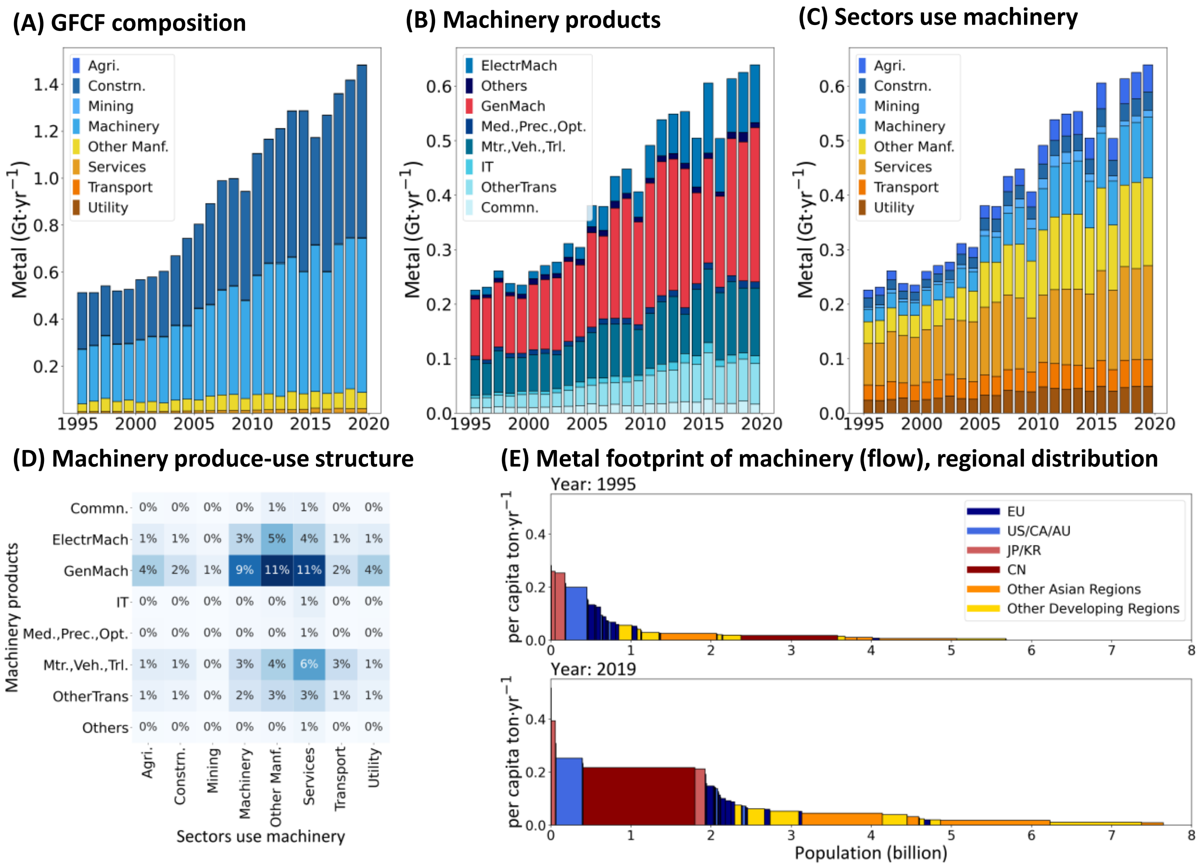

Figure S5 Metal Footprints of Machinery Production. (A) Metal footprint driven by Gross Fixed Capital Formation (GFCF): breakdown by product. Machinery parts are depicted in light blue. (B) The metal footprint of machinery production: breakdown by detailed machinery category. (C) The metal footprint of machinery production: breakdown by use sector. (D) Use the structure of detailed machinery products in 2019 (normalized by the total amount). (E) The metal footprint of machinery production in 1995 and 2019. The figures show the metal footprint of machinery production, broken down by product, category, use sector, and region. The featured region includes European countries (EU), the United States, Canada and Australia (US/CA/AU), Japan and Korea (JP/KR), China (CN), India (IN), rest of Asian countries (RoA), rest of Middle Eastern countries (RoM) and rest of African countries (RoF).

MachFP_Material/cap	Agriculture and Food	Construction	Extraction & Mining	Machinery	Other Manufacturing	Other Services	Transport	Utility	Total
CN	1.73	0.42	0.80	4.36	7.88	2.76	1.72	2.48	22.15
EU	1.57	1.12	0.27	1.36	3.16	7.40	1.29	1.71	17.89
JP_KR	1.60	3.00	0.10	6.82	6.22	11.85	2.69	1.98	34.26
OtherAsia	0.54	0.54	0.18	0.56	1.69	1.27	0.54	0.90	6.22
OtherDevelop	0.41	0.47	0.17	1.08	1.30	1.10	0.56	0.43	5.52
US_CA_AU	2.01	2.10	0.83	1.61	6.32	18.03	2.90	1.93	35.73

MachFP_GHG/cap	Agriculture and Food	Construction	Extraction & Mining	Machinery	Other Manufacturing	Other Services	Transport	Utility	Total
CN	0.87	0.21	0.42	2.14	4.23	1.40	1.03	1.51	11.83
EU	1.00	0.72	0.18	0.81	2.00	4.61	0.82	1.08	11.21
JP_KR	1.18	2.21	0.07	4.49	4.38	8.05	1.85	1.52	23.75
OtherAsia	0.27	0.26	0.09	0.28	0.85	0.64	0.27	0.46	3.13
OtherDevelop	0.23	0.25	0.09	0.57	0.75	0.61	0.31	0.27	3.09
US_CA_AU	1.28	1.32	0.55	1.04	3.93	11.15	1.72	1.26	22.26

MachFP_Metal/cap	Agriculture and Food	Construction	Extraction & Mining	Machinery	Other Manufacturing	Other Services	Transport	Utility	Total
CN	0.13	0.03	0.06	0.33	0.59	0.21	0.13	0.18	1.66
EU	0.15	0.11	0.03	0.12	0.29	0.67	0.13	0.15	1.65
JP_KR	0.25	0.43	0.02	0.94	0.89	1.44	0.31	0.33	4.61
OtherAsia	0.03	0.03	0.01	0.03	0.10	0.07	0.03	0.05	0.35
OtherDevelop	0.03	0.04	0.01	0.09	0.10	0.09	0.05	0.03	0.44
US_CA_AU	0.19	0.19	0.08	0.14	0.50	1.58	0.25	0.18	3.12

Figure S6. Per capita breakdown of material, carbon, and metal footprints for machinery stock, categorized by region and sector (ton/cap)

MachFP_Material/VA	Agriculture and Food	Construction	Extraction & Mining	Machinery	Other Manufacturing	Other Services	Transport	Utility
CN	2.27	0.42	6.19	4.71	5.35	0.82	4.14	20.69
EU	1.45	0.28	1.02	0.65	1.55	0.57	0.46	2.28
JP_KR	1.73	0.80	3.53	2.22	2.12	0.88	0.98	2.07
OtherAsia	1.55	1.65	1.44	3.58	5.36	1.25	2.43	7.18
OtherDevelop	1.02	0.80	0.69	4.39	3.65	0.62	1.47	3.93
US_CA_AU	2.11	0.27	0.92	0.71	2.83	0.68	0.53	1.59

MachFP_GHG/VA	Agriculture and Food	Construction	Extraction & Mining	Machinery	Other Manufacturing	Other Services	Transport	Utility
CN	1.14	0.21	3.21	2.31	2.87	0.42	2.50	12.62
EU	0.92	0.18	0.69	0.39	0.98	0.36	0.29	1.44
JP_KR	1.28	0.59	2.58	1.46	1.49	0.60	0.67	1.59
OtherAsia	0.77	0.80	0.72	1.78	2.70	0.64	1.23	3.68
OtherDevelop	0.58	0.43	0.38	2.32	2.11	0.35	0.82	2.45
US_CA_AU	1.35	0.17	0.61	0.46	1.76	0.42	0.32	1.04

MachFP_Metal/VA	Agriculture and Food	Construction	Extraction & Mining	Machinery	Other Manufacturing	Other Services	Transport	Utility
CN	0.17	0.03	0.47	0.36	0.40	0.06	0.31	1.53
EU	0.14	0.03	0.09	0.06	0.14	0.05	0.05	0.21
JP_KR	0.27	0.11	0.53	0.31	0.30	0.11	0.11	0.34
OtherAsia	0.09	0.09	0.08	0.20	0.30	0.07	0.14	0.40
OtherDevelop	0.08	0.06	0.05	0.35	0.29	0.05	0.12	0.31
US_CA_AU	0.20	0.02	0.09	0.06	0.22	0.06	0.05	0.15

Figure S7. Breakdown of per value-added material, carbon, and metal footprints for machinery stock, categorized by region and sector (ton/k EUR)**.** Industry-specific value added and deflator data for each year are sourced from EXIOBASE.

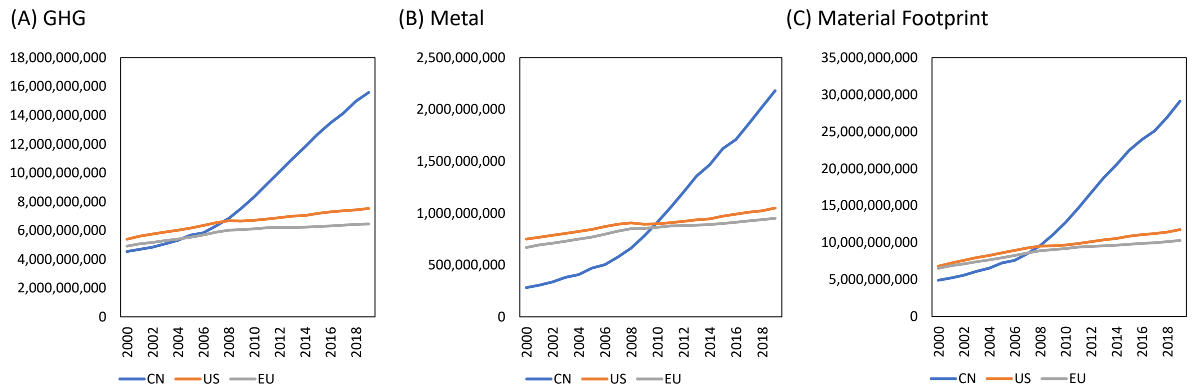

Figure S8. Carbon (GHG), Metal, and Material footprint of machinery stock of China, the United States, and European Countries.

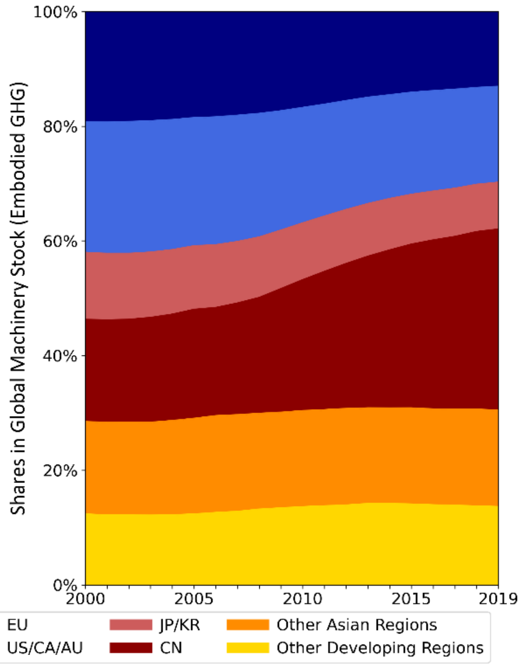

Figure S9. Shares of Carbon (GHG) Footprint Embodied in Machinery Stock

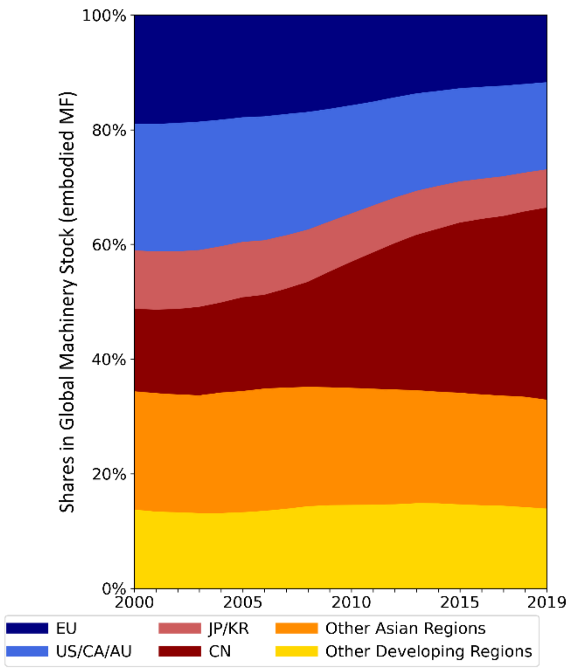

Figure S10. Shares of Material Footprint Embodied in Machinery Stock

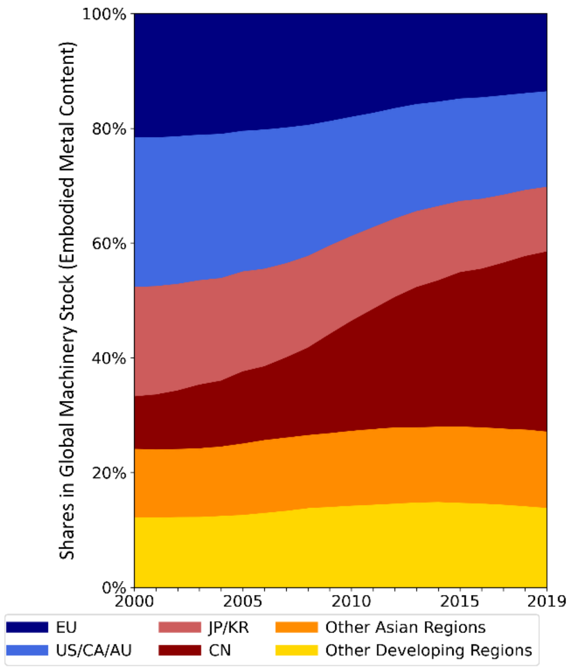

Figure S11. Shares of Metal Footprint Embodied in Machinery Stock

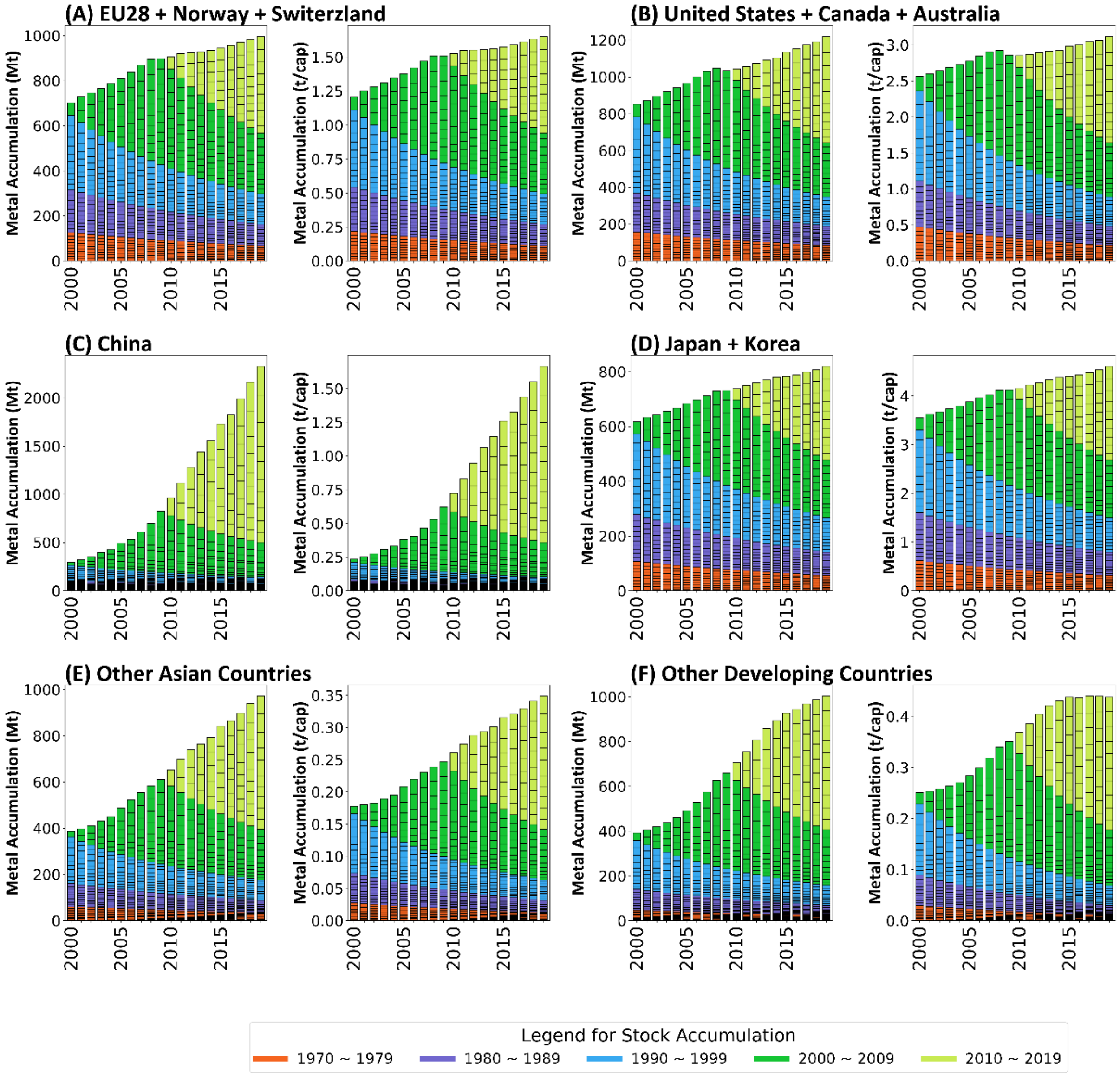

Figure S12. Metal Footprint embodied in Machinery Stock. (A) - (F) represent different region groups (see Supplementary Notes for grouping classification). The first and third columns indicate the metal footprint embodied in machinery stocks over the years, while the second and fourth columns indicate the per capita levels.

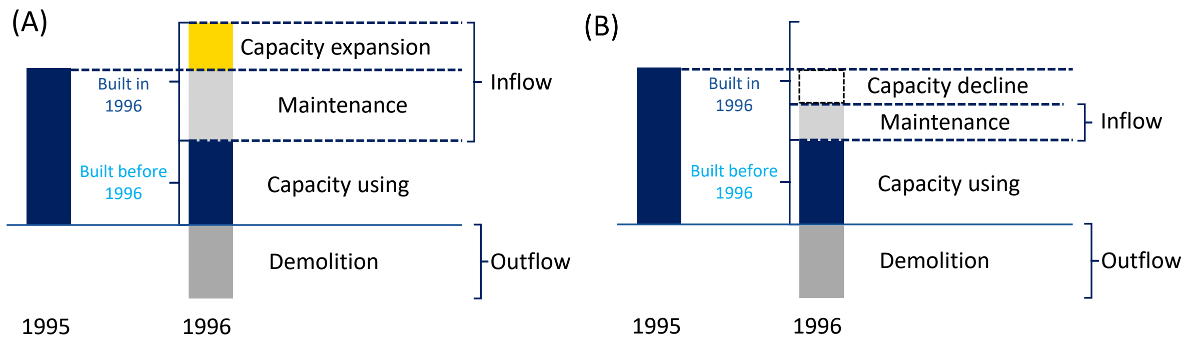

Figure S13. Overview of Inflow and outflow (e.g., demolition, maintenance, capacity expansion, and declines) of associated footprints

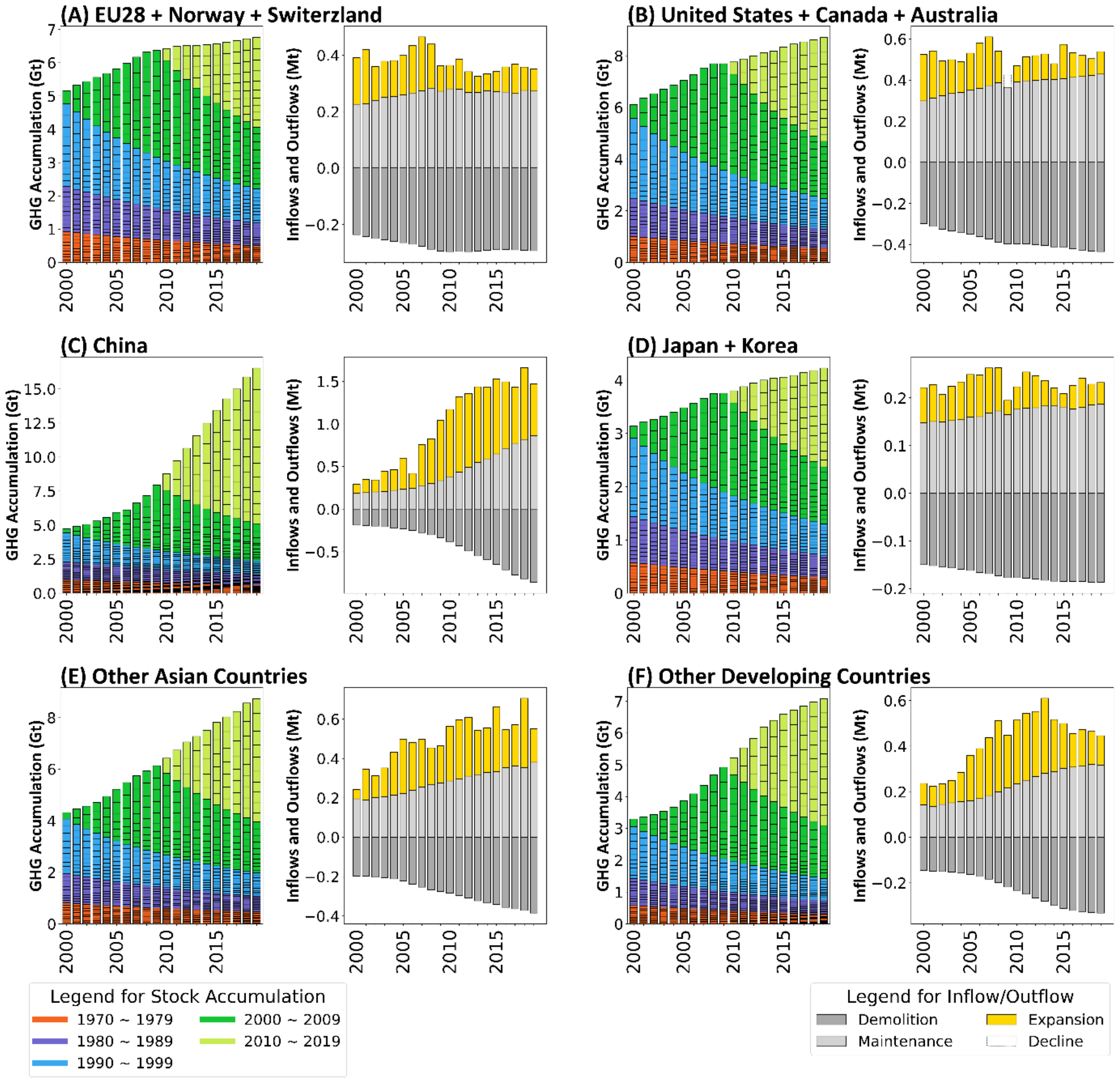

Figure S14. Carbon Footprint embodied in Machinery Stock and Inflows, and Outflows across Different Regions (survival curve approach). (A) - (F) represent different region groups (see Supplementary Notes for grouping classification). The first and third columns indicate the carbon footprint embodied in machinery stocks over the years, while the second and fourth columns indicate the inflows and outflows that include the parts of demolition, maintenance, expansion, and decline. For detailed definitions, refer to Fig.S13. Results were calculated using the survival curve.

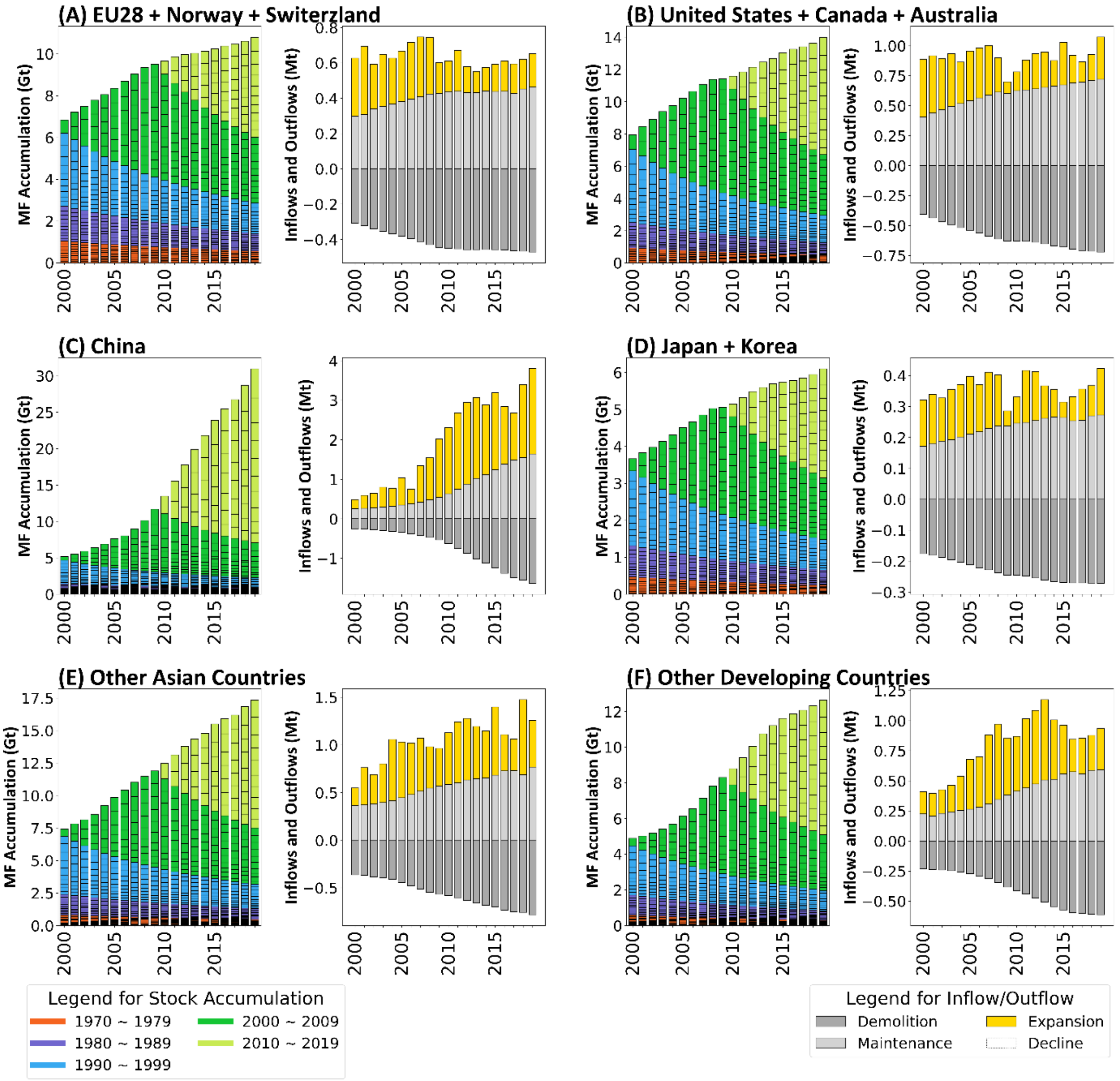

Figure S15. Material Footprint embodied in Machinery Stock and Inflows, and Outflows across Different Regions (survival curve approach). (A) - (F) represent different region groups (see Supplementary Notes for grouping classification). The first and third columns indicate the material footprint embodied in machinery stocks over the years, while the second and fourth columns indicate the inflows and outflows that include the parts of demolition, maintenance, expansion, and decline. For detailed definitions, refer to Fig.S13 in the Supplementary Information. Results were calculated using the survival curve.

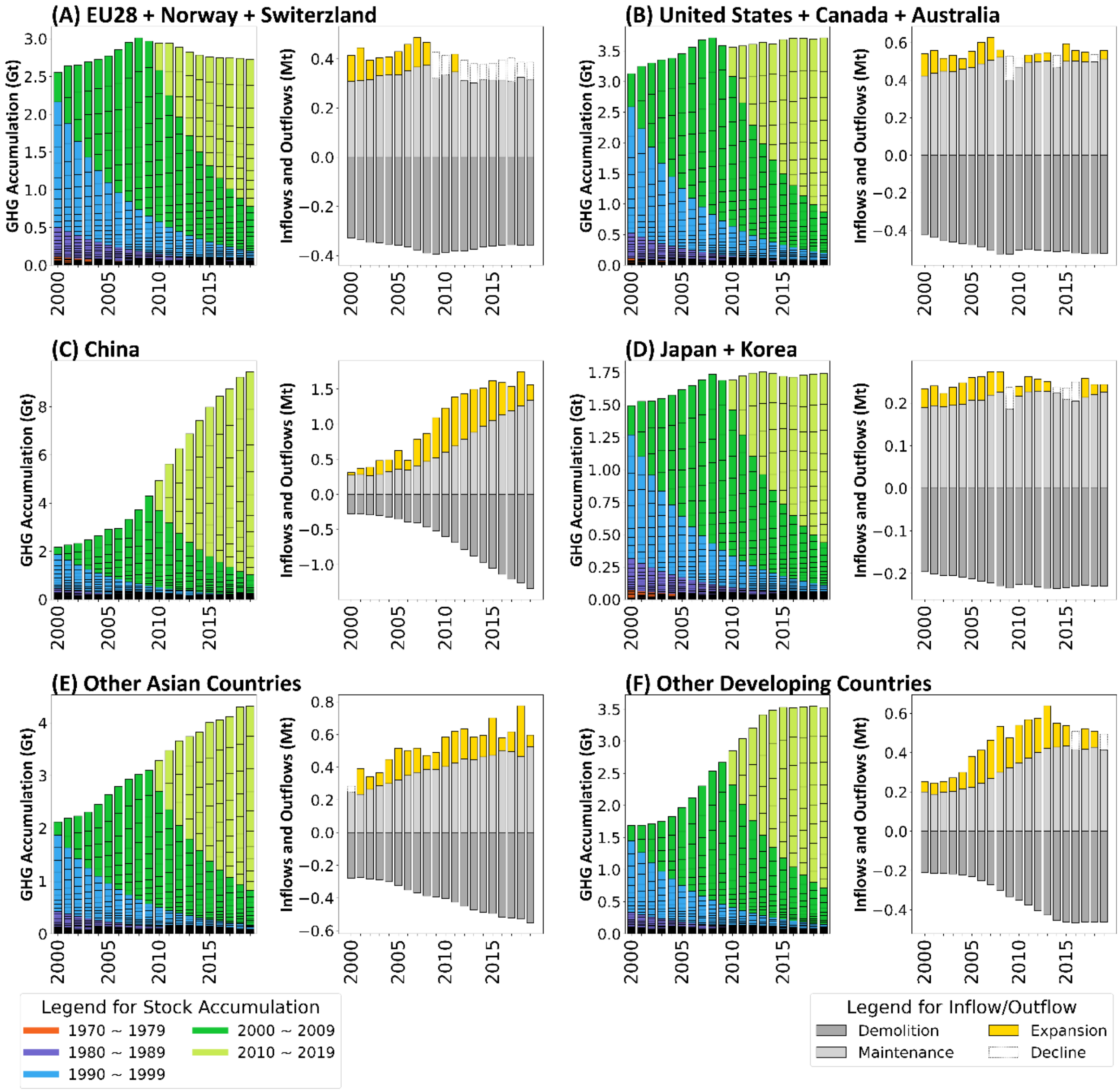

Figure S16. Carbon Footprint embodied in Machinery Stock and Inflows, and Outflows across Different Regions (depreciation approach). (A) - (F) represent different region groups (see Supplementary Notes for grouping classification). The first and third columns indicate the carbon footprint embodied in machinery stocks over the years, while the second and fourth columns indicate the inflows and outflows that include the parts of demolition, maintenance, expansion, and decline. For detailed definitions, refer to Fig.S13 in the Supplementary Information. Results were calculated using the depreciation rates.

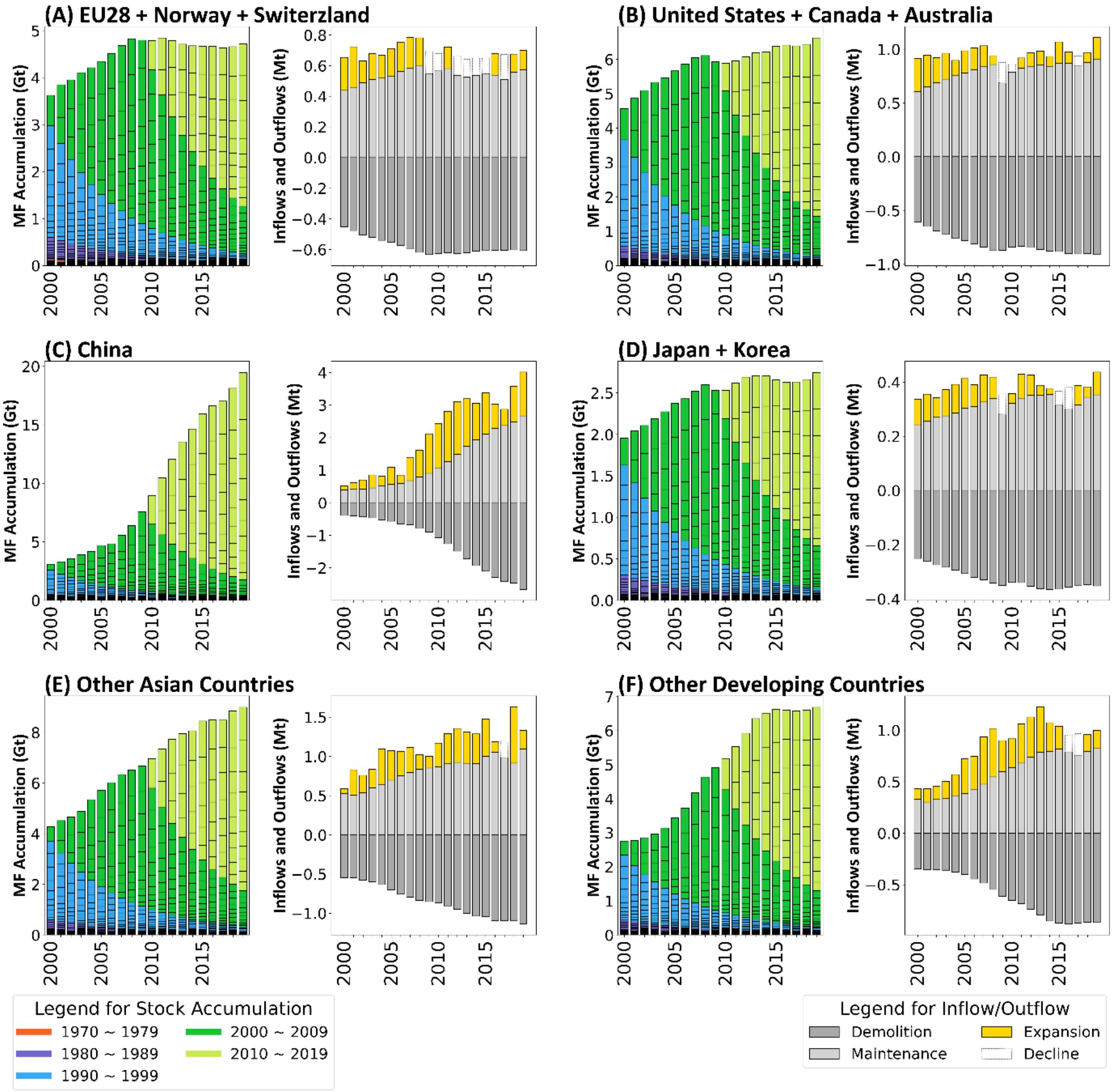

Figure S17. Material Footprint embodied in Machinery Stock and Inflows, and Outflows across Different Regions (depreciation approach). (A) - (F) represent different region groups (see Supplementary Notes for grouping classification). The first and third columns indicate the material footprint embodied in machinery stocks over the years, while the second and fourth columns indicate the inflows and outflows that include the parts of demolition, maintenance, expansion, and decline. For detailed definitions, refer to Fig.S13 in the Supplementary Information. Results were calculated using the depreciation rates.

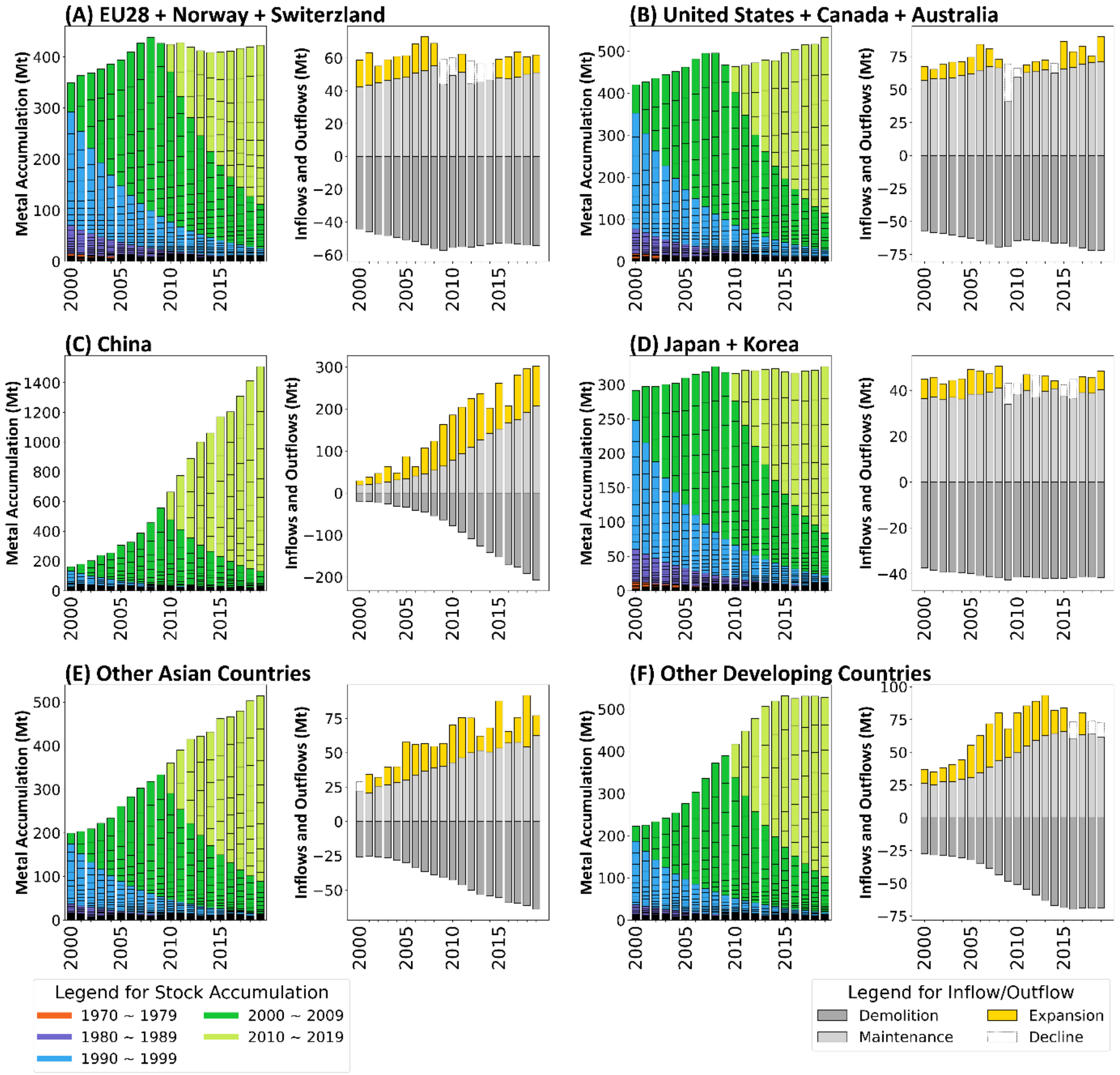

Figure S18. Metal Footprint embodied in Machinery Stock and Inflows, and Outflows across Different Regions (depreciation approach). (A) - (F) represent different region groups (see Supplementary Notes for grouping classification). The first and third columns indicate the metal footprint embodied in machinery stocks over the years, while the second and fourth columns indicate the inflows and outflows that include the parts of demolition, maintenance, expansion, and decline. For detailed definitions, refer to Fig.S13 in the Supplementary Information. Results were calculated using the <u>depreciation rates</u>.

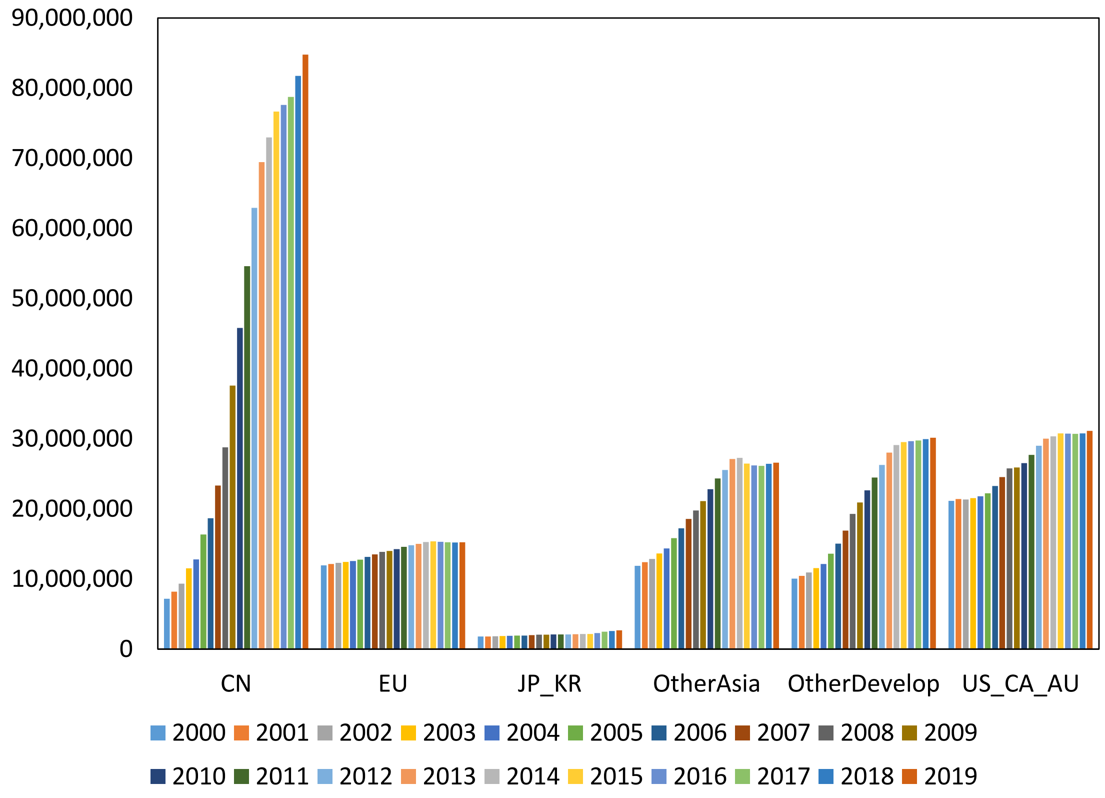

Figure S19. Metal Footprint Embodied in Machinery Stock in the Extraction & Mining Sector (ton).

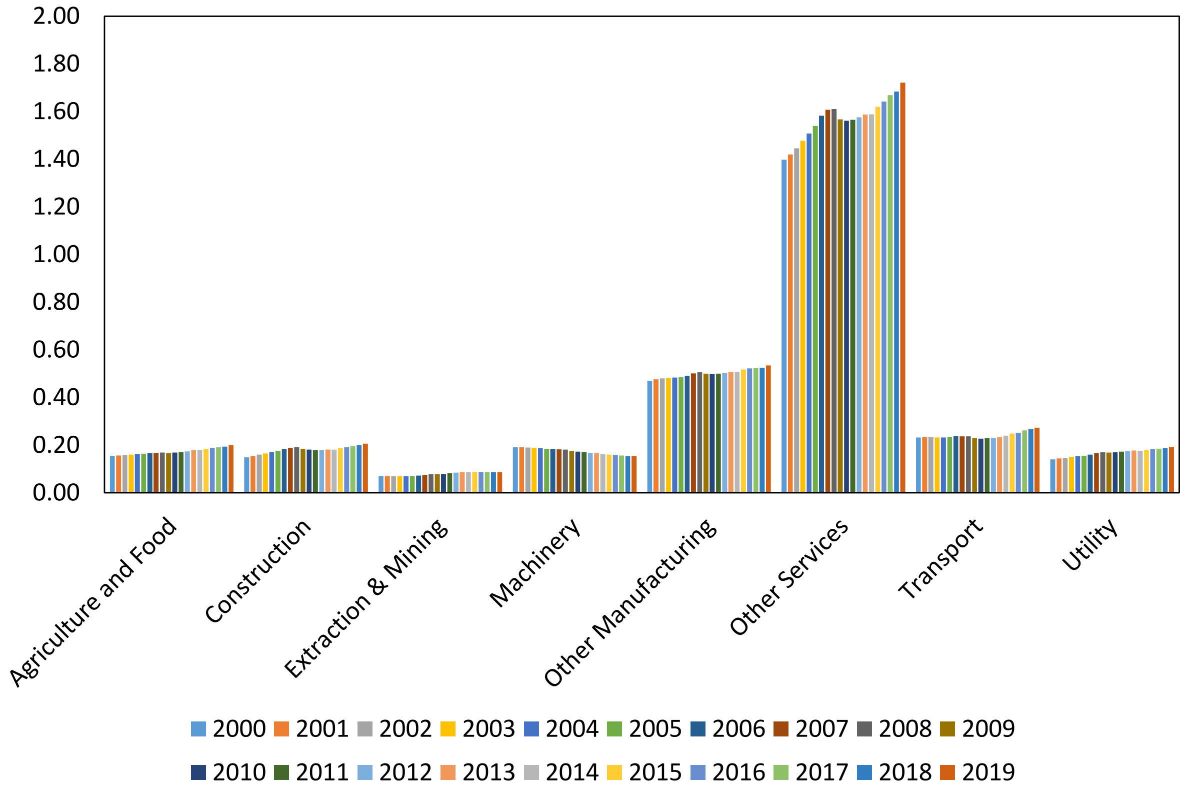

Figure S20. Metal Footprint Embodied in Machinery Stock in the United States (ton/cap).

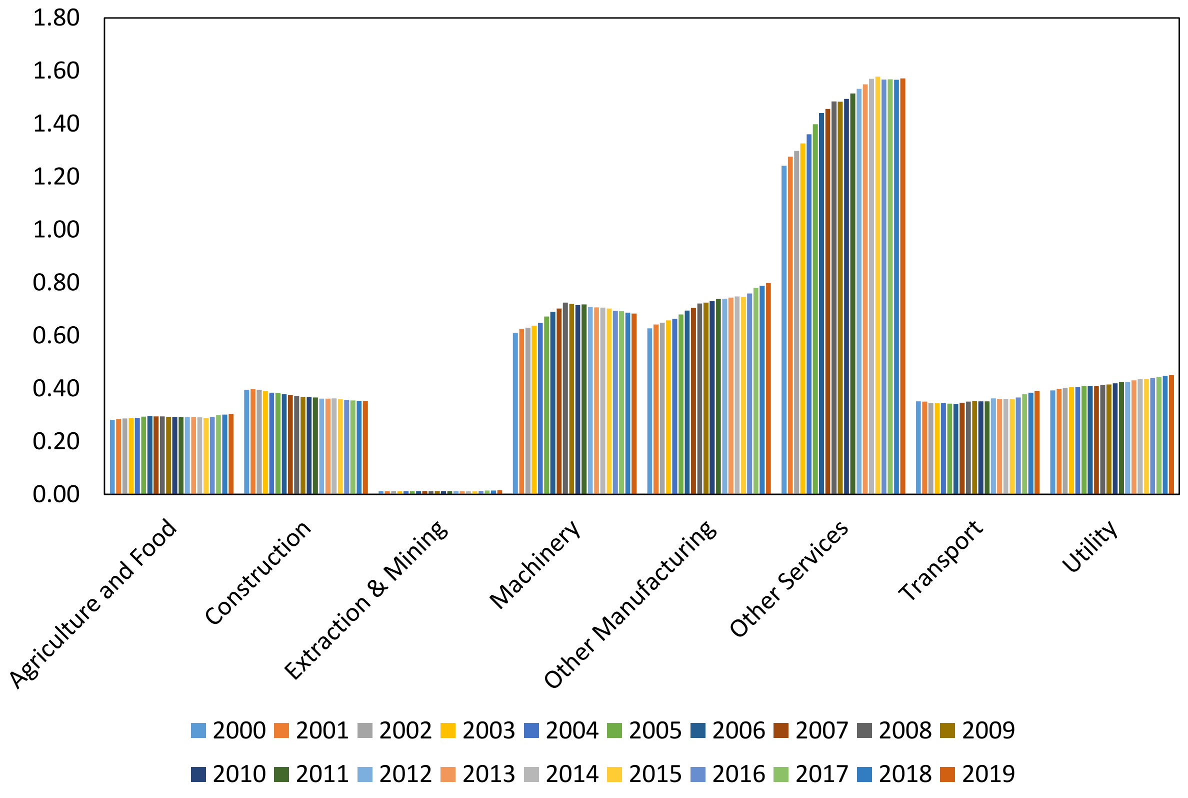

Figure S21. Metal Footprint Embodied in Machinery Stock in Japan (ton/cap)**.**

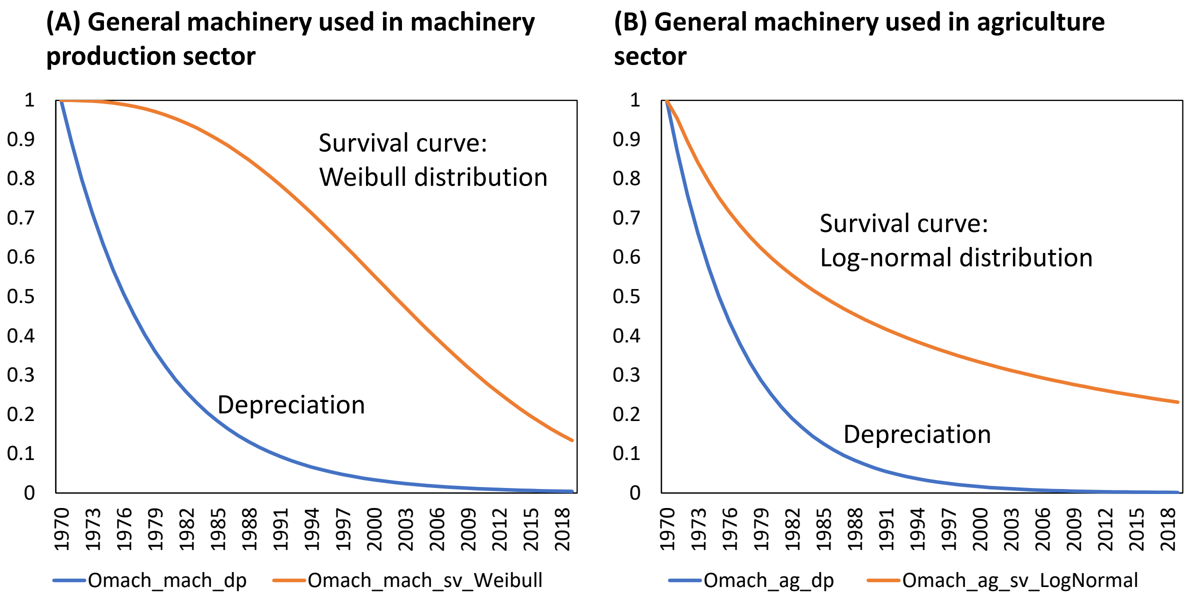

Figure S22 Survival curve and the curve derived from the depreciation: (A) the general machinery (assets) used in the machinery production sector; (B) the general machinery (assets) used in the agriculture sector.

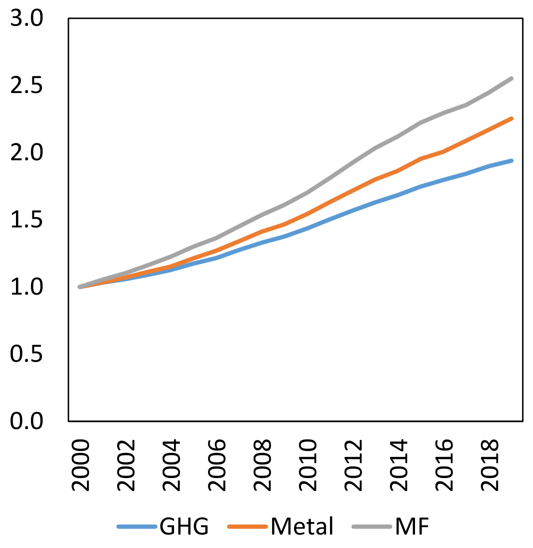

Figure S23 Growth of Global Carbon (GHG), Metal and Material Footprint (2000 = 1)

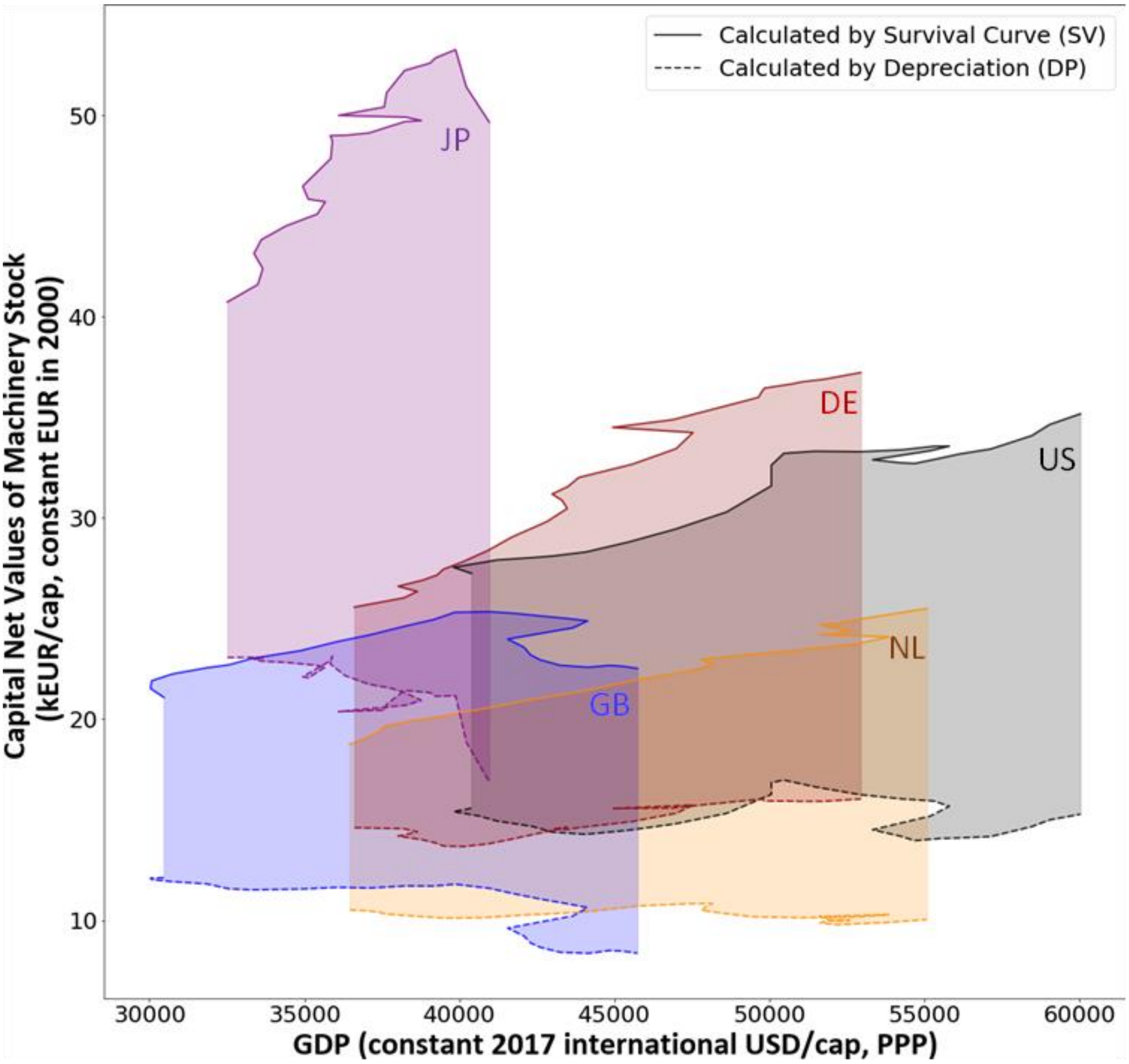

Figure S24. Capital Net Values of Machinery Stock (kEUR/cap vs. GDP/cap, PPP). The distribution of the capital is calculated using the survival curve (SV) and the curve derived from the depreciating rates (DP) for different countries. Solid lines depict the results based on the survival curve, while the dotted lines below indicate outcomes derived from depreciation rates. The shaded area highlights the discrepancy between the two approaches Featured countries include the United Kingdom (GB), the United States (US), the Netherlands (NL), Germany (DE), and Japan (JP).

Supplemental Tables

Table S1 Country List and Aggregation

No	Individual economies	abbreviation	Region group
1	Austria	AT	EU
2	Belgium	BE	EU
3	Bulgaria	BG	EU
4	Cyprus	CY	EU
5	Czech Republic	CZ	EU
6	Germany	DE	EU
7	Denmark	DK	EU
8	Estonia	EE	EU
9	Spain	ES	EU
10	Finland	FI	EU
11	France	FR	EU
12	Greece	GR	EU
13	Croatia	HR	EU
14	Hungary	HU	EU
15	Ireland	IE	EU
16	Italy	IT	EU
17	Lithuania	LT	EU
18	Luxembourg	LU	EU
19	Latvia	LV	EU
20	Malta	MT	EU
21	Netherlands	NL	EU
22	Poland	PL	EU
23	Portugal	PT	EU
24	Romania	RO	EU

**25**	Sweden	SE	EU
**26**	Slovenia	SI	EU
**27**	Slovakia	SK	EU
**28**	United Kingdom	GB	EU
**29**	United States	US	US_CA_AU
**30**	Japan	JP	JP_KR
**31**	China	CN	CN
**32**	Canada	CA	US_CA_AU
**33**	South Korea	KR	JP_KR
**34**	Brazil	BR	OtherDevelop
**35**	India	IN	OtherAsia
**36**	Mexico	MX	OtherDevelop
**37**	Russia	RU	OtherAsia
**38**	Australia	AU	US_CA_AU
**39**	Switzerland	CH	EU
**40**	Turkey	TR	OtherDevelop
**41**	Taiwan	TW	CN
**42**	Norway	NO	EU
**43**	Indonesia	ID	OtherAsia
**44**	South Africa	ZA	OtherDevelop
**45**	RoW Asia and Pacific	WA	OtherAsia
**46**	RoW America	WL	OtherDevelop
**47**	RoW Europe	WE	EU
**48**	RoW Africa	WF	OtherDevelop
**49**	RoW Middle East	WM	OtherDevelop

Table S2 Statistical results on value added vs. footprint embodied in machinery stocks

Types of footprints	sector	R-square	elasticity	intercept
**GHG**	Agriculture and Food	0.99	0.46***	1.69***
**GHG**	Extraction & Mining	0.99	0.59***	0.41***
**GHG**	Other Manufacturing	0.95	1.14***	-4.22***
**GHG**	Machinery	0.97	0.77***	-0.71***
**GHG**	Utility	0.89	1.26***	-5.30***
**GHG**	Construction	0.97	0.80***	-0.57***
**GHG**	Transport	0.97	1.95***	-10.81***
**GHG**	Other Services	0.98	0.94***	-1.96***
**metal**	Agriculture and Food	0.98	0.33***	3.12***
**metal**	Extraction & Mining	0.99	0.46***	1.92***
**metal**	Other Manufacturing	0.97	0.79***	-0.40***
**metal**	Machinery	0.97	0.61***	1.26***
**metal**	Utility	0.90	0.87***	-1.20***
**metal**	Construction	0.97	0.61***	1.59***
**metal**	Transport	0.96	1.15***	-2.94***
**metal**	Other Services	0.99	0.73***	0.63***
**MF**	Agriculture and Food	0.99	0.36***	2.54***
**MF**	Extraction & Mining	0.99	0.49***	1.14***
**MF**	Other Manufacturing	0.95	0.82***	-1.46***
**MF**	Machinery	0.98	0.66***	0.16***
**MF**	Utility	0.89	0.86***	-2.07***
**MF**	Construction	0.97	0.65***	0.69***
**MF**	Transport	0.97	1.24***	-4.88***
**MF**	Other Services	0.98	0.73***	-0.20***

***, p-value<0.01

Table S3 Data source of aggregated capital flow and depreciation

No	Name	region	Aggregated Capital Flow (Structure)	Dataset for Depreciation	Period	Notes
**1**	Austria	AT	EUKlems	EUKlems	1995-2017	
**2**	Belgium	BE	EUKlems	EUKlems	1995-2017	
**3**	Bulgaria	BG	EUKlems	EUKlems	1995-2017	Machinery is in the aggregated the resolution
**4**	Cyprus	CY	EUKlems	EUKlems	1995-2017	Machinery is in the aggregated the resolution
**5**	Czech Republic	CZ	EUKlems	EUKlems	1995-2017	
**6**	Germany	DE	EUKlems	EUKlems	1995-2017	
**7**	Denmark	DK	EUKlems	EUKlems	1995-2017	
**8**	Estonia	EE	EUKlems	EUKlems	1995-2017	
**9**	Spain	ES	EUKlems	EUKlems	1995-2017	
**10**	Finland	FI	EUKlems	EUKlems	1995-2017	
**11**	France	FR	EUKlems	EUKlems	1995-2017	
**12**	Greece	GR	EUKlems	EUKlems	1995-2017	Machinery is in the aggregated the resolution
**13**	Croatia	HR	EU's structure	EUKlems	1995-2017	Only depreciation
**14**	Hungary	HU	EUKlems	EUKlems	1995-2017	
**15**	Ireland	IE	EUKlems	EUKlems	1995-2017	Machinery is in the aggregate

						d the resolution
**16**	Italy	IT	EUKlems	EUKlems	1995-2017	
**17**	Lithuania	LT	EUKlems	EUKlems	1995-2017	
**18**	Luxembourg	LU	EUKlems	EUKlems	1995-2017	
**19**	Latvia	LV	EUKlems	EUKlems	1995-2017	
**20**	Malta	MT	EUKlems	EUKlems	1995-2017	Machinery is only available from 2011-2017
**21**	Netherlands	NL	EUKlems	EUKlems	1995-2017	
**22**	Poland	PL	EUKlems	EUKlems	1995-2017	
**23**	Portugal	PT	EUKlems	EUKlems	1995-2017	Machinery is only available from 2010-2016
**24**	Romania	RO	EUKlems	EUKlems	1995-2017	
**25**	Sweden	SE	EUKlems	EUKlems	1995-2017	
**26**	Slovenia	SI	EUKlems	EUKlems	1995-2017	
**27**	Slovakia	SK	EUKlems	EUKlems	1995-2017	
**28**	United Kingdom	GB	EUKlems	EUKlems	1995-2017	
**29**	United States	US	EUKlems	EUKlems	1970-2017	
**30**	Japan	JP	EUKlems	EUKlems	1995-2017	
**31**	China	CN	Statistical Yearbook of the Chinese Investment in Fixed Assets[6]	Literature estimation[10, 11]	1995-2017	
**32**	Canada	CA	Statistics Canada[8]	US's structure	1995-2019	https://www150.statcan.gc.ca/t1/tbl1/en

						/tv.action?pid=3610009901
**33**	South Korea	KR	World KLEMS	World KLEMS	1995-2012	
**34**	Brazil	BR	Mexico's structure	Mexico's structure	1995-2018	
**35**	India	IN	Government of India, Ministry of Statistics and Programme Implementation[9]	Global average	2011-2019	http://164.100.161.63/publication/national-accounts-statistics-2012
**36**	Mexico	MX	LA Klems	LA Klems	1995-2018	
**37**	Russia	RU	Average of China and EU's structure	Global average	1995-2017	
**38**	Australia	AU	Average of the structure of EU, UK, and US	World KLEMS	1995-2017	
**39**	Switzerland	CH	EU's structure	EU's structure	1995-2017	
**40**	Turkey	TR	Average of China and EU's structure	Global average	1995-2017	
**41**	Taiwan, China	TW	Mainland China's structure	Mainland China's structure	1995-2017	
**42**	Norway	NO	Statistics Norway[7]	EU's structure	1995-2019	https://www.ssb.no/en/statbank/table/11189
**43**	Indonesia	ID	Average of China, India, and EU's structure	Global average	1995-2017	
**44**	South Africa	ZA	Average of China, Mexico, and EU's structure	Global average	1995-2017	

**45**	RoW Asia and Pacific	WA	Average of China, India, and EU's structure	Global average	1995-2017
**46**	RoW America	WL	Mexico's structure	Mexico's structure	1995-2018
**47**	RoW Europe	WE	EU's structure	EU's structure	1995-2017
**48**	RoW Africa	WF	Average of China, Mexico, and India's structure	Global average	1995-2017
**49**	RoW Middle East	WM	Average of China and EU's structure	Global average	1995-2017

Table S4 Comparison of our results with similar studies

Description in other studies	Values (source)	Our estimates on similar flows	Description of our values
**[Steel][2008][Global] The global proportion of steel used in vehicles and industrial equipment compared to its total use in all end-use products**	29%[12]		41%
**[Steel][2001][China] Steel used for transport and equipment in intermediate industries compared to total steel consumption in those industries, China, 2011**	26%[13]		29%
**[Steel][2007][North America] Steel used for transport and equipment in intermediate industries compared to total steel consumption in those industries, North America, 2011**	43%[13]		53%
**[Steel][2007][Global] Steel used for transport and equipment in intermediate industries compared to total steel consumption in those industries, World, 2011**	27%[13]	Within the same time frame and location scope, the metal footprint (primary metal) is driven by machinery-related products (Table 1)	40%
**[Steel][1995-2011][China] End-use shares of China's steel use in other machinery & appliances, Electrical machinery & appliances, Transportation and Furniture; other manufactured goods n.e.c. sectors.**	30%-47%[14, 15]		28%-39%
**[Steel][1995-2011][India] End-use shares of India's steel use in other machinery & appliances, Electrical machinery & appliances, Transportation and Furniture; other manufactured goods n.e.c. sectors.**	48%-64%[6]		20%-57%
**[Steel][1995-2011][UK] End-use shares of UK's steel use in other machinery & appliances, Electrical machinery & appliances, Transportation and Furniture; other manufactured goods n.e.c. sectors.**	61-76%[6]		41%-57%
**[Iron][2000][Global] Global iron entering use for transport equipment and industrial machinery, 2000**	44%[16]		42%

**[Aluminum][2007][Global] The global proportion of Aluminum used in vehicles and industrial equipment compared to its total use in all end-use products.**	47%[17]		41%
**[Copper][2012][China] Copper ore footprint driven by manufacturing sector compared to total copper ore footprint, China, 2012**	44%[18]		34%

Reference

(1) Rincon-Aznar, A.; Riley, R.; Young, G. *Academic review of asset lives in the UK*; 2017. https://www.niesr.ac.uk/wp-content/uploads/2021/10/DP474.pdf (accessed 2022-04-27).

(2) Dirk, v. d. B.; Mark, d. H.; Ron, d. H.; Myriam, H. *Measuring capital in the Netherlands*; Statistics Netherlands, The Hague, 2009. https://www.cbs.nl/-/media/imported/documents/2009/31/2009-36-x10-pub-en.pdf (accessed 2022-04-27).

(3) National Bank of Belgium. *Computing capital stock in the Belgian national accounts according to the ESA 2010 - Methodological note*; National Bank of Belgium, Belgium, 2014. https://www.nbb.be/doc/dq/e_method/m_sec2010d2_en.pdf (accessed 2022-04-27).

(4) Erumban, A. A. Lifetimes of machinery and equipment: evidence from Dutch manufacturing. *Review of Income and Wealth* **2008**, *54* (2), 237-268. DOI: https://doi.org/10.1111/j.1475-4991.2008.00272.x.

(5) Stehrer, R.; Bykova, A.; Jäger, K.; Reiter, O.; Schwarzhappel, M. *Industry level growth and productivity data with special focus on intangible assets - Statistical Report*; Vienna Institute for International Economic Studies Vienna, 2019. https://euklems.eu/wp-content/uploads/2019/10/Methodology.pdf (accessed 2022-10-13).

(6) Statistical Department of Fixed Assets Investment. *China Fixed Assets Investment Statistical Yearbook*; National Bureau of Statistics of China, Beijing, 1995-2018. (accessed 2023-08-13).

(7) Statistics Norway. *Gross fixed capital formation and capital stocks, by asset type and industry 1970 - 2020*; Statistics Norway, Oslo, 2022. https://www.ssb.no/en/statbank/table/11189 (accessed 2022-03-14).

(8) Statistics Canada. *Flows and stocks of fixed non-residential capital, by sector of industry and type of asset, Canada*; Statistics Canada, Ottawa, 2022. https://www150.statcan.gc.ca/t1/tbl1/en/tv.action?pid=3610009701 (accessed 2023-12-01).

(9) Ministry of Statistics and Programme Implementation of India. *National Accounts Statistics*; Ministry of Statistics and Programme Implementation of India, New Delhi, 2012-2019. https://164.100.161.63/download-reports (accessed 2022-03-18).

(10) Linlin Sun; Jiao, J. Capital Stock Estimation of Chinese Industries Based on Endogenous Depreciation Rate. *Journal of Beijing University of Aeronautics and Astronautics (Social Sciences Edition)* **2016**, *29* (3), 97-107. DOI: https://doi.org/10.13766/j.bhsk.1008-2204.2016.0063.

(11) Wei Wang; Jie Chen; Mao, S. Revaluation of Chinese Capital Stocks Based on Ten Categories:1978-2016. *Journal of Quantitative & Technical Economics* **2017**, *10*, 60-77. DOI: http://10.13653/j.cnki.jqte.2017.10.004.

(12) Cullen, J. M.; Allwood, J. M.; Bambach, M. D. Mapping the Global Flow of Steel: From Steelmaking to End-Use Goods. *Environmental Science & Technology* **2012**, *46* (24), 13048-13055. DOI: 10.1021/es302433p.

(13) Aguilar-Hernandez, G. A.; Deetman, S.; Merciai, S.; Rodrigues, J. F. D.; Tukker, A. Global distribution of material inflows to in-use stocks in 2011 and its implications for a circularity transition. *Journal of Industrial Ecology* **2021**, *25* (6), 1447-1461. DOI: https://doi.org/10.1111/jiec.13179.

(14) Streeck, J.; Wieland, H.; Pauliuk, S.; Plank, B.; Nakajima, K.; Wiedenhofer, D. A review of methods to trace material flows into final products in dynamic material flow analysis: Comparative application of six methods to the United States and EXIOBASE3 regions, Part 2. *Journal of Industrial Ecology* **2023**, *27* (2), 457-475. DOI: https://doi.org/10.1111/jiec.13379.

(15) Pauliuk, S.; Wang, T.; Müller, D. B. Moving Toward the Circular Economy: The Role of Stocks in the Chinese Steel Cycle. *Environmental Science & Technology* **2012**, *46* (1), 148-154. DOI: 10.1021/es201904c.

(16) Wang, T.; Müller, D. B.; Graedel, T. E. Forging the Anthropogenic Iron Cycle. *Environmental Science & Technology* **2007**, *41* (14), 5120-5129. DOI: 10.1021/es062761t.

(17) Cullen, J. M.; Allwood, J. M. Mapping the Global Flow of Aluminum: From Liquid Aluminum to End-Use Goods. *Environmental Science & Technology* **2013**, *47* (7), 3057-3064. DOI: 10.1021/es304256s.

(18) Liu, L.; Schandl, H.; West, J.; Jiang, M.; Ren, Z.; Chen, D.; Zhu, B. Copper ore material footprints and transfers embodied in domestic and international trade of provinces in China. *Journal of Industrial Ecology* **2022**, *26* (4), 1423-1436. DOI: https://doi.org/10.1111/jiec.13285.
